# Streptomyces castrisilvae sp. nov. and Streptomyces glycanivorans sp. nov., novel soil streptomycetes metabolizing mutan and alternan

**DOI:** 10.1099/ijsem.0.006514

**Published:** 2024-09-12

**Authors:** Tove Widén, Albert Tafur Rangel, Vincent Lombard, Elodie Drula, Scott Mazurkewich, Nicolas Terrapon, Eduard J. Kerkhoven, Johan Larsbrink

**Affiliations:** 1 Department of Life Sciences, Chalmers University of Technology, SE-412 96 Gothenburg, Sweden; 2 Novo Nordisk Foundation Center for Biosustainability, Technical University of Denmark, DK-2800 Kgs. Lyngby, Denmark; 3 Architecture et Fonction des Macromolécules Biologiques, USC 1408 INRAE, UMR 7257 AMU, CNRS, FR-13288 Marseille, France; 4 INRAE, Biodiversité et Biotechnologie Fongiques, UMR 1163, Aix Marseille Université, Marseille, France; 5 Wallenberg Wood Science Center, Chalmers University of Technology, SE-412 96, Gothenburg, Sweden

**Keywords:** alpha-glucan, alternan, carbohydrate-active enzymes, mutan, *Streptomyces*, whole-genome sequencing

## Abstract

Six bacterial strains, Mut1^T^, Mut2, Alt1, Alt2, Alt3^T^, and Alt4, were isolated from soil samples collected in parks in Gothenburg, Sweden, based on their ability to utilize the insoluble polysaccharides α−1,3-glucan (mutan; Mut strains) or the mixed-linkage α−1,3/α−1,6-glucan (alternan; Alt strains). Analysis of 16S rRNA gene sequences identified all strains as members of the genus *Streptomyces*. The genomes of the strains were sequenced and subsequent phylogenetic analyses identified Mut2 as a strain of *Streptomyces laculatispora* and Alt1, Alt2 and Alt4 as strains of *Streptomyces poriferorum,* while Mut1^T^ and Alt3^T^ were most closely related to the type strains *Streptomyces drozdowiczii* NBRC 101007^T^ and *Streptomyces atroolivaceus* NRRL ISP-5137^T^, respectively. Comprehensive genomic and biochemical characterizations were conducted, highlighting typical features of *Streptomyces*, such as large genomes (8.0–9.6 Mb) with high G+C content (70.5–72.0%). All six strains also encode a wide repertoire of putative carbohydrate-active enzymes, indicating a capability to utilize various complex polysaccharides as carbon sources such as starch, mutan, and cellulose, which was confirmed experimentally. Based on phylogenetic and phenotypic characterization, our study suggests that strains Mut1^T^ and Alt3^T^ represent novel species in the genus *Streptomyces* for which the names *Streptomyces castrisilvae* sp. nov. and *Streptomyces glycanivorans* sp. nov. are proposed, with strains Mut1^T^ (=DSM 117248^T^=CCUG 77596^T^) and Alt3^T^ (=DSM 117252^T^=CCUG 77600^T^) representing the respective type strains.

## Introduction

*Streptomyces* is the largest genus in the phylum *Actinomycetota* with over 700 taxa with validly published name to date [1]. *Streptomyces* species are aerobic, filamentous Gram-positive bacteria that generally have large genomes with high G+C content [2]. They are especially known for their ability to produce secondary metabolites including many clinically important antibiotics such as streptomycin [3]. *Streptomyces* species inhabit diverse environments and are abundant in soil, where they fulfil an important role as nutrient recyclers thanks to their ability to decompose various types of biomass, from plants and fungi to insect and animal tissues [4,5]. For many species, the enzymatic machinery for utilization of abundant polysaccharides, such as cellulose, xylan and chitin, have been extensively studied [6]. For instance, enzymes with interesting properties, such as an alkaline-tolerant chitinase [7], a thermostable xylanase [8], and a cellulase tolerant to the presence of detergents [9], have been found in different *Streptomyces* species.

Carbohydrate-active enzymes (CAZymes) are categorized in the CAZy database (www.cazy.org [10]). The abundance of putative degradative CAZymes encoded in the large genomes of *Streptomyces* species suggests an ability to also metabolize a wide range of less studied polysaccharides [2,10], such as mutan (α−1,3-glucan) and alternan (mixed-linkage α−1,3/α−1,6-glucan). Both are insoluble exopolysaccharides found in dental plaque biofilms where they are produced by bacteria such as streptococci [11,12]. Mutan is also present as a structural component in the cell walls of some fungi and is therefore prevalent in nature and has the potential of being used as an energy source. For both polysaccharides, there is limited information regarding enzymatic degradation. Mutan hydrolases (mutanases) have been discovered in glycoside hydrolase families 71 and 87 (GH71 and GH87, respectively) [10], but only a handful of enzymes from each family have been characterized [13,15]. From the genus *Streptomyces*, one mutanase from *Streptomyces thermodiastaticus* HF 3-3 [16] and one mycodextranase (hydrolysing mixed-linkage α−1,3/1,4-glucans) from *Streptomyces* sp. J-13–3 [17] have previously been identified and investigated, where the former has also been structurally determined using X-ray crystallography (PDB ID 7C7D). In contrast, no enzymes able to efficiently cleave alternan (alternanases) have been identified as of today from any organism. However, dextranases have in some cases been reported to act weakly on alternan, though this activity was assumed to stem from hydrolysis of regions of the substrate with consecutive α−1,6-linkages [18,19].

The aim of this study was to discover and characterize soil bacteria able to metabolize either mutan, alternan, or both polysaccharides. We were able to isolate six *Streptomyces* strains from park soils in Gothenburg, Sweden, using selective culturing conditions, and these were physiologically and genomically characterized. All strains exhibited broad abilities to metabolize complex polysaccharides, which was attributed to large numbers of CAZymes encoded in their genomes. Based on the comparisons of genomic sequences, three of the strains were found to belong to the species *Streptomyces poriferorum* [20] and one to *Streptomyces laculatispora* [21]. The two remaining strains were found to be previously uncharacterized species, and for these we propose the names *Streptomyces castrisilvae* sp. nov. and *Streptomyces glycanivorans* sp. nov.

## Isolation

Soil samples were collected from two parks in Gothenburg, Sweden (Slottsskogen: 57° 41′ 23.8″ N 11° 57′ 02.0″ E, soil containing sawdust-like residues from a nearby dead tree, and 57° 41′ 12.3″ N 11° 57′ 00.6″ E, mossy soil between the roots of a maple tree, and Kungsparken: 57° 42′ 02.3″ N 11° 57′ 50.0″ E, soil beneath a beech tree in proximity to an unidentified mushroom (Fig. S1, available in the online version of this article) on 2 August and 9 November 2022, respectively. One gram of soil was suspended in 10 ml sterile water and plated in serial dilutions on M9 minimal medium agar plates (disodium hydrogen phosphate 6.778 g l^–1^, potassium phosphate 3 g l^–1^, ammonium chloride 1 g l^–1^, sodium chloride 0.5 g l^–1^, magnesium sulphate 0.24 g l^–1^, calcium dichloride 0.011 g l^–1^) with 4 g l^–1^ of either of the polysaccharides mutan or alternan as the sole carbon source. Mutan and alternan were synthesized enzymatically as previously described [22]. Briefly, genes encoding the glycosyltransferases GtfJ and GtfL (Uniprot: Q00600_STRSL, Q55264_STRSL) from *Streptococcus salivarius* ATCC 25975 were cloned from genomic DNA into pET28a vectors, the genes expressed in *Escherichia coli* λDE3, and the resulting recombinant proteins purified using immobilized metal ion affinity chromatography on an ÄKTA system (GE Healthcare). Of either enzyme, GtfJ for mutan and GtfL for alternan production, 10 mg were added to 1 l sucrose (1 M) solution containing 10 mM sodium phosphate at pH 6 and incubated at room temperature for 5 days, after which the water-insoluble mutan and alternan were collected and washed with water through centrifugation or filtration, respectively. The polysaccharides were then lyophilized and stored at 4 °C until usage. The inoculated mutan- or alternan-containing plates were incubated at 20 °C until substantial growth could be detected, after 5–6 days. Isolates were then selected for further investigation based on the presence of a clearing zone of the opaque polysaccharides around the colonies (see example in Fig. S2). Two isolates, Mut1^T^ and Mut2, were selected from the mutan-containing plates, with the soil samples originating from the two sites in Slottsskogen. Four isolates, Alt1, Alt2, Alt3^T^, and Alt4, were selected from the alternan-containing plates which all originated from Kungsparken. To ensure the purity of the strains, colonies were re-streaked first on M9 media with mutan or alternan as the sole carbon source and then on lysogeny broth until only one type of colony could be seen. Colonies of the strains were used as templates for PCR amplifications of the 16S rRNA gene sequence regions using the primers 27F (5′-AGAGTTTGATCMTGGCTCAG-3′) and 1492R (5′-CGGTTACCTTGTTACGACTT-3′) and DreamTaq DNA Polymerase (Thermo Fisher Scientific) according to the manufacturer’s instructions. The resulting PCR products were sent for Sanger sequencing (Eurofins Genomics) and the sequences then submitted to 16S RefSeq Nucleotide sequence records with the accession numbers as follows: Mut1^T^, PP532741; Mut2, PP532742; Alt1, PP532743; Alt2, PP532744; Alt3^T^, PP532745; Alt4, PP532746. Sequences were compared to the 16S rRNA sequences database using blast [23], identifying all isolates as *Streptomyces* species. The strains were routinely grown on International *Streptomyces* Project (ISP) 2 medium (yeast extract 4 g l^–1^, malt extract 10 g l^–1^, glucose 4 g l^–1^, agar 20 g l^–1^) and maintained as glycerol stocks (20%) at −80 °C.

## Genome sequencing and statistics

DNA extraction, genome sequencing, and genome assembly for all strains were performed by DNASense (Aalborg Denmark). DNA was extracted using the DNeasy PowerSoil Pro Kit following the manufacturer’s recommendations (Qiagen). The extracted DNA was subsequently subjected to cleanup using DNeasy PowerClean Pro cleanup kit. DNA concentrations and purities were measured with the Qubit dsDNA HS Assay kit (Thermo Fisher Scientific) using a NanoDrop One (Thermo Fisher Scientific). DNA size distributions were evaluated using the Genomic DNA ScreenTapes on an Agilent Tapestation 4200 (Agilent). Barcoded SQK-RBK114.96 and SQK-LSK114.24 libraries were prepared according to the manufacturer’s protocol, except for minor modifications (Oxford Nanopore Technologies). The library was loaded onto a primed FLO-PRO114M (R10.4.1) flow cell and sequenced on a PromethION P2 Solo device with MinKNOW Release 22.07.5 (MinKNOW Core 5.3.0-rc6-p2solo). FAST5 data were basecalled using the Super-accurate basecalling algorithm (Guppy version 6.3.8). Adapters were removed with Porechop version 0.2.4 and low-quality reads were filtered using Filtlong version 0.2.1. Basic read statistics including amount of data produced, median read quality and read N50 were assessed with NanoStat version1.6.0 [24].

Draft *de novo* assemblies were produced with Flye version 2.9.1-b1780 [25] and subsequently polished twice with Medaka version 1.7.2 (Oxford Nanopore Technologies). Assembly graphs and draft assembly coverage were visualized and inspected with Bandage version 0.8.1 [26]. Each genome assembly was quality-assessed with CheckM version 1.1.3 [27]. Assembled genomes were classified with the GTDBtk version 2.1.1 [28] against the Genome Taxonomy Database (GTDB) [29]. All the strains were classified as belonging to the genus *Streptomyces*, and only Mut2 was species-level classified (*S. laculatispora* [21]); nevertheless, taxonomy check done by NCBI reports it as inconclusive. Bakta version 1.6.1 was used to annotate the genomes using the –compliant flag (Genbank/ENA/DDJB compliance) [30]. rRNA genes were extracted using Barrnap. Notably, Alt3^T^ yielded limited initial sequencing data, prompting multiple rounds of DNA extraction and sequencing. To address this, multiple independent DNA extracts were sequenced for all strains, not just Alt3^T^, until achieving satisfactory coverage for Alt3^T^. This process accounts for the exceptionally high coverage reported for the other samples. As is typical with *Streptomyces* species, all isolated strains had large genomes, 8.0–9.6 Mb, and high G+C content, 72% for Mut1^T^ and 70.5% for all other strains, which is within the expected range of 67.0–78.0 % as described for the genus *Streptomyces* [31,32]. Genome features are shown in Table 1.

**Table 1.** Genome features of the isolated and sequenced *Streptomyces* strains compared with the representative genome of the most closely related species Mut1^T^ is most closely related to *S. drozdowiczii*, Mut2 to *S. laculatispora*, Alt1, Alt2 and Alt4 to *S. poriferorum*, and Alt3^T^ to *S. atroolivaceus*. Note that a discrepancy between assembly and genome size is due to the presence of plasmids. n.d., No data.

Genome features	Mut1^T^	Mut2	Alt1	Alt2	Alt3^T^	Alt4	*S. drozdowiczii* MDA8-470*	*S. laculatispora* NRRL B24909^T^ [20]*	*S. poriferorum* P01-B04^T^ [20]*	*S. atroolivaceus* NRRL ISP-5137^T^*
Assembly size (Mb)	8	8.6	9.5	9.6	8.7	9.6	7.4	7.3	8.9	8.2
Genome size (Mb)	7.6	8.5	9.1	9.5	8.4	n.d.	7.4	7.3	8.9	8.2
G+C content (%)	72	70.5	70.5	70.5	70.5	70.5	72	70.6	70.7	70.5
Mean depth (fold)	469	459	448	451	64	412	250	120	59	232
Contigs	4	5	2	2	5	3	1	663	695	106
Read N50 (bp)	6590	8258	14 637	11 101	7445	12 569	n.d.	n.d.	n.d.	n.d.
Contig N50 (Mb)	7.6	8.50	9.1	9.5	8.4	7.3	7.4	0.0147	0.026	0.3321
Contig L50	1	1	1	1	1	1	1	158	99	8
Completeness estimation (%)	99.91	99.19	100	100	99.19	100	91.73	83.96	96.53	99.56
Contamination estimation (%)	1.07	0.38	0	0.38	0	0.38	1.14	0.95%	0.09	0
Genes	6849	7665	8237	8422	7716	8384	6709	6863	8095	7323
Protein-coding	6671	7091	7951	8127	7362	8098	6031	6453	7715	6971
rRNA genes	18	18	21	18	21	18	18	n.d.	3	n.d.
tRNA genes	72	74	73	78	76	78	66	59	66	n.d.
Genome assembly accession number (GCA_*)	030719295.1	030719275.1	030719255.1	030719235.1	030719215.1	030732165.1	026167665.1	017353455.1	019399235.1	000717025.1
Genome accession number	CP120997.1	CP120992.1	CP120990.1	CP120988.1	CP120983.1	JARVXU000000000	CP098740.1	JAFMPZ000000000	JAELVH000000000	JNXG00000000
Assembly level	Complete genome	Complete genome	Complete genome	Complete genome	Complete genome	Contig level (3)	Complete genome	Scaffold level	Scaffold level	Contig level

*information from the NCBI assembly page of the listed genome assembly accession number [51] (accessed 28 May 28, 2024).

## Phylogeny

**Fig. 1.**
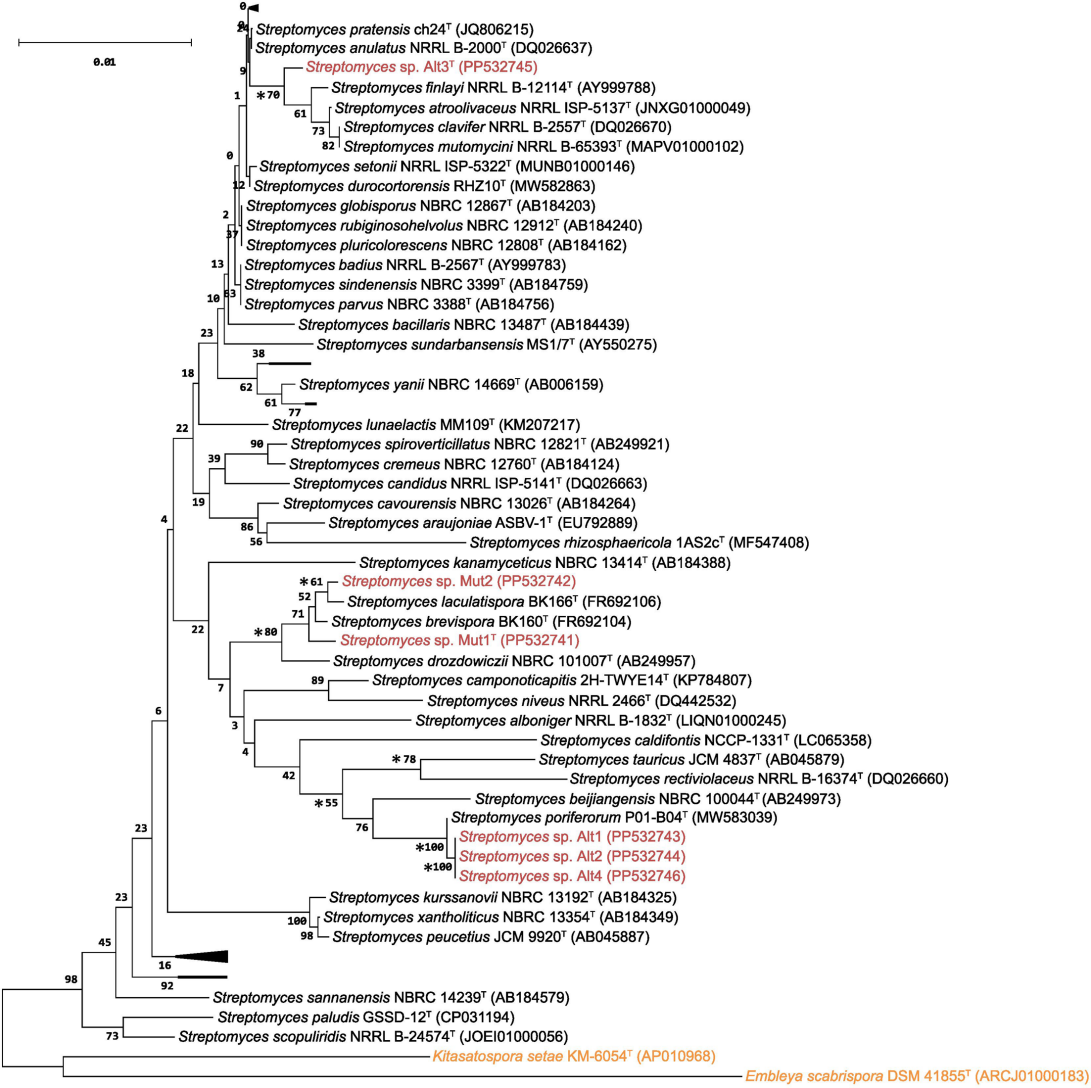
Phylogenetic tree of *Streptomyces* strains Mut1^T^, Mut2, Alt1, Alt2, Alt3^T^, and Alt4 based on 16S rRNA gene sequences. The tree was reconstructed using the neighbour-joining method. The root position of the neighbour-joining tree was determined using *Kitasatospora setae* KM-6054^T^ (AP010968) and *Embleya scabrispora* DSM 41855^T^ (ARCJ01000183) as the outgroups. Bold numbers on the branches are the confidence limits estimated by bootstrap analysis with 1000 replicates. Blank spaces represent collapsed nodes; for full version see Fig. S3. The strains investigated in this study are shown in red, and strains in orange represent outgroups. Asterisks indicate branches also recovered in the maximum-likelihood and maximum-parsimony trees.

Since only strain Mut2 was classified on the species level, and to validate the classification done against the GTDBtk, 16S rRNA gene sequences of strains Alt1, Alt2, Alt3^T^, Alt4, Mut1^T^, and Mut2 were compared with those of known bacterial species using EzBioCloud database [33]. The highest pairwise sequence similarity match for strains Alt1, Alt2, and Alt4 was *S. poriferorum* P01-B04^T^ (100%) [20], for Alt3^T^, *Streptomyces atroolivaceus* NRRL ISP-5137^T^ (99.78%), for Mut1^T^, *Streptomyces brevispora* BK160^T^ (99.85%), and for Mut2, *S. laculatispora* BK166 ^T^ (98.70%). The optimal tree was inferred using the neighbour-joining method [34] (Fig. 1) using mega version 11 [35], based on the 16S rRNA genes sequences of unique species obtained from similarity search for each strain studied here (up to 50 hits), yielding 72 *Streptomyces* species (similarity >97.3 %) [36]. The percentage of replicate trees in which the associated taxa clustered together were assessed by bootstrap analysis (1000 replicates) [37]. The evolutionary distances were computed using the maximum composite likelihood method [38] and are in the units of the number of base substitutions per site. This analysis involved 80 nucleotide sequences. All ambiguous positions were removed for each sequence pair (pairwise deletion option). There were a total of 1515 positions in the final dataset. Strains Alt1, Alt2, and Alt4 are most closely related to *S. poriferorum* P01-B04^T^ and this was supported by a 100% bootstrap value (Fig. 1). Strain Mut1^T^ is closely related to *S. brevispora* BK160^T^ and *S. laculatispora* BK166^T^, while strain Mut2 is most closely related to *S. laculatispora* BK166^T^, though with a low bootstrap value of 61 % and low resolution. Strain Alt3^T^ is closely related to *Streptomyces mutomycini* NRRL B-65393^T^, *Streptomyces clavifer* NRRL B-2557^T^, *S. atroolivaceus* NRRL ISP-5137^T^ and *Streptomyces finlayi* NRRL B-12114^T^. Similar clustering was also seen in phylogenetic trees constructed by the maximum-likelihood and maximum parsimony methods (Fig. S4).

**Fig. 2.**
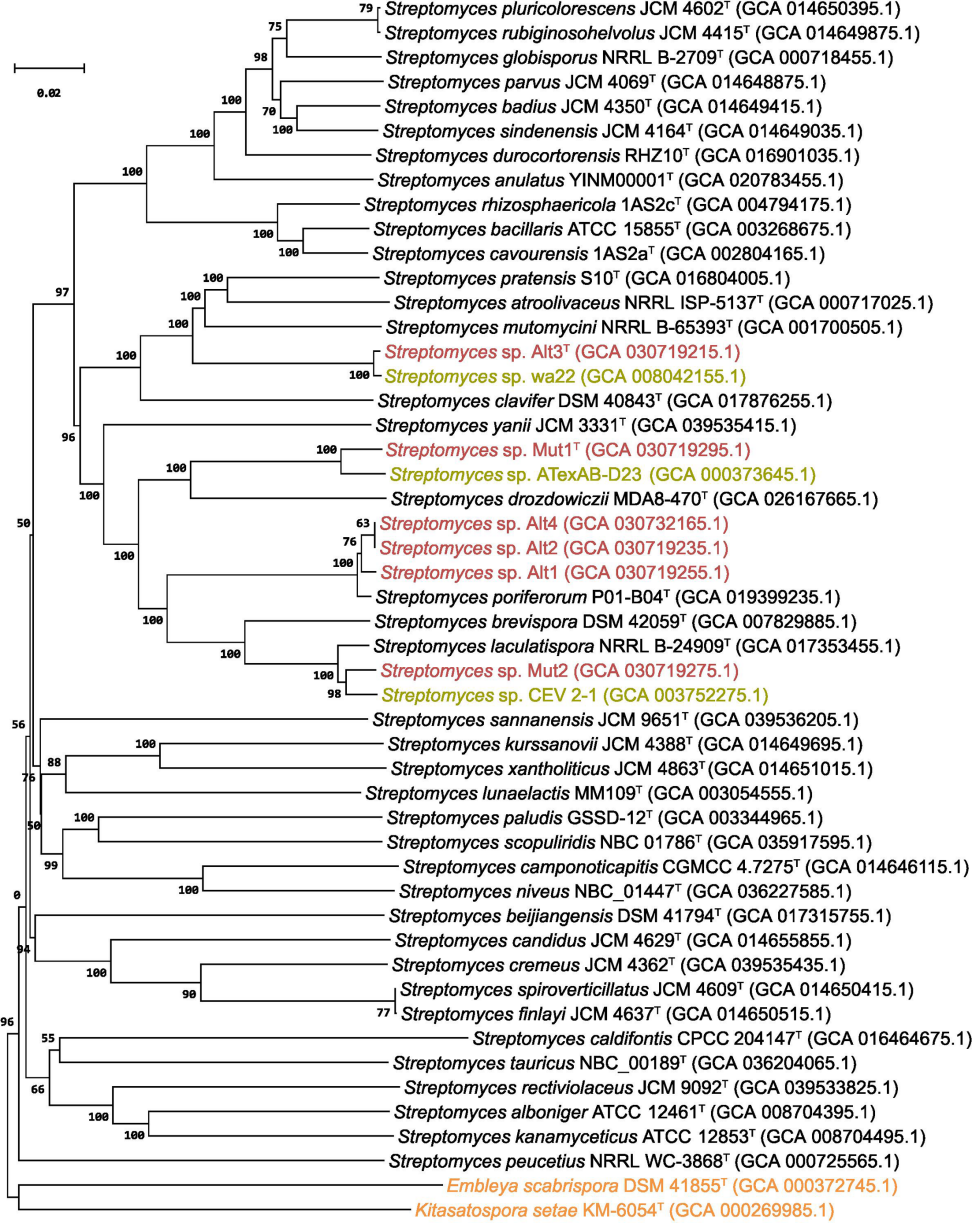
Phylogenetic tree of *Streptomyces* strains Mut1^T^, Mut2, Alt1, Alt2, Alt3^T^, and Alt4, from genome sequences. The tree was inferred with FastME 2.1.6.1 [52] from GBDP distances calculated by TYGS from whole-genome sequences. The branch lengths (decimal numbers) are scaled in terms of GBDP distance formula d_5_. The numbers above branches are GBDP pseudo-bootstrap support values>60 % from 100 replications, with an average branch support of 89.9 %. The strains investigated in this study are shown in red, the closest strains without validly published name in green, and strains in orange represent outgroups. The tree was rooted at the outgroup strains.

In an effort to further support the observed phylogeny, we complemented the 16S rRNA-based phylogram (Fig. 1) with a whole-genome sequence-based analysis (Fig. 2). The genome sequences were uploaded to the Type (strain) Genome Server (TYGS; https://tygs.dsmz.de) [39]. The query was restricted to the species analysed in the 16S rRNA phylogenetic tree (Fig. 1), except *Streptomyces* sp. wa22 (GCA_008042155.1), *Streptomyces* sp*.* ATexAB-D23 (GCA_000373645.1), *Streptomyces* sp*.* CEV 2–1 (GCA_003752275.1), which were also included as they were identified as the most similar strains to Alt3^T^, Mut1^T^, and Mut2, respectively, in an initial study with non-restricted queries on TYGS. For the phylogenomic inference, all pairwise comparisons among the set of genomes were conducted using the Genome blast Distance Phylogeny approach (GBDP) and accurate intergenomic distances inferred under the algorithm 'trimming' and distance formula d_5_ [40] with 100 distance replicates calculated for each. The results showed good agreement with the 16S rRNA gene sequence phylogeny, showing 37 species clusters. The provided query strains were assigned to four of these clusters, one comprising Alt1, Alt2, and Alt4, together with *S. poriferorum* [20]*,* and one cluster for Alt3^T^, Mut1^T^, and Mut2 individually, identifying them as potentially representing new species.

While useful for determination of genus and the relationship between species, 16S rRNA gene sequences do not always have sufficient resolution to identify and distinguish between species, and this is the case for *Streptomyces* [41,43]. To validate potential species designations of the new isolated strains, overall genomic relatedness indices (OGRI) were assessed through the calculation of average nucleotide identity (ANI) and digital DNA–DNA hybridization (dDDH) values. The dDDH values and confidence intervals were calculated using the recommended settings of the Genome-to-Genome Distance Calculator 4.0 [40,44]. For ANI comparisons, each new isolate strain was analysed against the most closely related strains based on the 16S rRNA gene sequence and genome based phylogeny. A total of 240 pairwise comparisons were done for each sequence using JSpecies Web Server (https://jspecies.ribohost.com/jspeciesws/), with the MUMmer approach [45]. A minimum threshold of 96.7% ANI was considered as being the same species [46], resulting in a total of seven comparisons ranked with an ANI score >97%, clustered in four groups (Table 2). These results are consistent with >70% dDDH values (Table 2), demonstrating that the strains have been reported previously. Based on the OGRI, Mut2 is a strain of *S. laculatispora* (sharing ANIm of 98.07% and dDDH of 81.7% with strain BK166^T^) and closely related to the unclassified *Streptomyces* strain CEV 2-1 (sharing ANIm of 98.25% and dDDH of 84.3%), and Alt1, Alt2 and Alt4 are *S. poriferorum* (sharing ANIm of 99.12, 99.15 and 99.15%, and dDDH of 92.1, 92.1 and 92.1% with strain P01-B04^T^, respectively). Furthermore, Mut1^T^ and *Streptomyces*. sp. AtexAB-D23 [47] as well as Alt3^T^ and *Streptomyces* sp. wa22 [48] are two previously unclassified and uncharacterized distinct *Streptomyces* species. Strain Mut1^T^ shared the highest OGRI with *Streptomyces drozdowiczii* NBRC 101007^T^ (ANIm of 90.12% and dDDH of 37.1%), while strain Alt3^T^ shared the highest OGRI with *S. atroolivaceus* NRRL ISP-5137^T^ (ANIm of 89.98% and dDDH 37.2%). Generally, differences in G+C content were very low for strains of the same species and higher when comparing between different species (Table 2).

**Table 2.** Pairwise comparison of the isolated *Streptomyces* strains and their closest sequenced strains

Species 1	Species 2	dDDH	G+C difference	ANIm
*Streptomyces* sp. Mut1^T^ (GCA_030719295.1)	*Streptomyces* sp. ATexAB-D23 (GCA_000373645.1)	78.2	0.02	97.69
*Streptomyces* sp. Mut1^T^ (GCA_030719295.1)	*Streptomyces drozdowiczii* NBRC 101007^T^ (GCA_026167665.1)	37.1	0.21	90.12
*Streptomyces* sp. Mut1^T^ (GCA_030719295.1)	*Streptomyces laculatispora* BK166^T^ (GCA_017353455.1)	32.8	1.13	88.65
*Streptomyces* sp. Mut1^T^ (GCA_030719295.1)	*Streptomyces brevispora* BK160^T^ (GCA_007829885.1)	32.3	1.43	88.57
*Streptomyces* sp. Mut2 (GCA_030719275.1)	*Streptomyces* sp. CEV 2–1 (GCA_003752275.1)	84.3	0.02	98.25
*Streptomyces* sp. Mut2 (GCA_030719275.1)	*Streptomyces laculatispora* BK166^T^ (GCA_017353455.1)	81.7	0.11	98.07
*Streptomyces* sp. Mut2 (GCA_030719275.1)	*Streptomyces brevispora* BK160^T^ (GCA_007829885.1)	49.2	0.19	93.22
*Streptomyces* sp. Mut2 (GCA_030719275.1)	*Streptomyces drozdowiczii* NBRC 101007^T^ (GCA_026167665.1)	30.5	1.45	87.89
*Streptomyces* sp. Alt1 (GCA_030719255.1)	*Streptomyces poriferorum* P01-B04^T^ (GCA_019399235.1)	92.1	0	99.12
*Streptomyces* sp. Alt2 (GCA_030719235.1)	*Streptomyces poriferorum* P01-B04^T^ (GCA_019399235.1)	92.1	0.05	99.15
*Streptomyces* sp. Alt4 (GCA_030732165.1)	*Streptomyces poriferorum* P01-B04^T^ (GCA_019399235.1)	92.1	0.04	99.15
*Streptomyces* sp. Alt3^T^ (GCA_030719215.1)	*Streptomyces* sp. wa22 (GCA_008042155.1)	97.1	0.25	99.68
*Streptomyces* sp. Alt3^T^ (GCA_030719215.1)	*Streptomyces atroolivaceus* NRRL ISP-5137^T^ (GCA_000717025.1)	37.2	0.18	89.98
*Streptomyces* sp. Alt3^T^ (GCA_030719215.1)	*Streptomyces anulatus* NRRL B-2000^T^ (GCA_020783455.1)	26.6	1.08	86.63
*Streptomyces* sp. Alt3^T^ (GCA_030719215.1)	*Streptomyces finlayi* NRRL B-12114^T^ (GCA_014650515.1)	22.5	0.78	85.43

Collectively, these analyses indicate that Alt1, Alt2 and Alt4 are strains of *S. poriferorum* and that Mut2 is a strain of *S. laculatispora*, while Mut1^T^ and Alt3^T^ appear to be new species in the genus *Streptomyces*.

## Genome features

The genome sequences for Mut1^T^ and Alt3^T^ were submitted to the antiSMASH server [49] to identify potential gene clusters encoding machineries to produce secondary metabolites, and a total of 24 and 33 clusters were predicted, respectively (Tables S1 and S2). The most common types of predicted secondary metabolites were polyketides followed by nonribosomal peptides (NRPs). In Alt3^T^, eight of the predicted clusters had a similarity >90% to known gene clusters producing either NRP, polyketide, or other known metabolites. In contrast, in Mut1^T^ only three clusters had a similarity >90% to known clusters, and these were in fact 100% identical to clusters known to produce coelichelin (NRP), ectoine (other), and venezuelin (post-translationally modified peptide; RiPP: lanthipeptide).

We next analysed the presence of putative CAZymes encoded by the strains. All encoded over 200 CAZymes in total, and especially glycoside hydrolases (GHs) and glycosyltransferases (GTs) were abundant. Overall, more CAZymes were found in the Alt- compared to the Mut strain genomes (Table 3). The degradative CAZymes (non-GTs) were further categorized by what substrates they were predicted to target, from individual plant or microbial glycan to more complex matrices (Table 4). All strains appear to encode enzymes to target most major polysaccharides from plants as well as microorganisms, and in particular they all encode large numbers of putative chitinases. General differences between the strains are that the Alt strains appear to have more enzymes for metabolism of pectins compared to the Mut strains. Alt1, Alt2, and Alt4, as expected had very similar CAZyme profiles, and were the only strains encoding putative fructanases.

**Table 3.** Number of CAZymes in the characterized strains, separated into CAZy classes

Enzyme class	Mut1^T^	Mut2	Alt1	Alt2	Alt3^T^	Alt4
Glycoside hydrolases	155	145	186	183	156	172
Glycosyltransferases	54	43	44	43	58	41
Polysaccharide lyases	7	13	18	16	16	14
Carbohydrate esterases	14	13	20	20	22	20
Auxiliary activities	6	4	6	6	5	6
Total	236	218	274	268	257	253

**Table 4.** Number of CAZymes in respective genomes targeting different polysaccharides The CAZymes were annotated by CAZy using semi-manual curation. All annotated genomes are available on CAZy, except for Alt4 as it is categorized as a contig-level genome. Note that for polyspecific families, the main activity of the family was chosen as target polysaccharide.

Target polysaccharide	CAZy families identified in genomes	Mut1^T^	Mut2	Alt1	Alt2	Alt3^T^	Alt4
Animal-/proteoglycan*	GH16_24, GH31_18, GH33, GH38, GH59, GH84, GH85, GH89, GH99, GH101, GH109, GH110, GH136, PL8, PL8_2, PL33_2	10	20	17	15	11	15
Arabinogalactan	GH30_5, GH43_24, GH154	1	2	4	4	1	4
Cellulose	GH5_1, GH5_2, GH6, GH9, GH12, GH48, GH51_3	8	4	9	10	6	11
Chitin	AA10, CE4, CE9, CE14, GH18, GH19_2, GH20, GH46, GH75	41	31	40	40	29	40
Fructan	GH32	0	0	4	4	0	3
Laminarin	GH16_3, GH55, GH64, GH158	8	6	8	8	7	8
(galacto-gluco-)Mannan	GH5_8, GH5_18, GH5_19, GH26, GH27, GH36, GH130_11	6	8	3	3	5	3
Microbial polysaccharide^†^	GH23, GH25, GH30_3, GH43_5, GH49, GH76, GH87, GH92, GH114, GH171, GH178, PL6_2, PL31, PL41	25	21	27	26	25	26
Starch	GH4, GH13 (many subfamilies), GH15, GH31_1, GH31_17, GH65, GH77, GH97	25	20	26	26	23	25
Pectin	GH28, GH35, GH42, GH43_5, GH43_26, GH53, GH78, GH93, GH106, CE8, CE12, PL1, PL3, PL9, PL11, PL26, PL42	12	8	22	22	25	23
Xylan	CE1, CE2, CE4, CE7, GH10, GH11, GH30, GH43, GH43_10, GH43_12, GH51_1, GH51_2	9	7	16	16	24	16
Xyloglucan	GH29, GH31_3, GH74, GH95	6	11	13	11	8	12
Unspecified β-glycan, β-glycoside	GH5, GH5_51, GH1, GH2, GH3	20	23	25	25	19	25

*Proteoglycans, e.g. hyaluronan and chondroitin sulfatesulphate, as well as blood/milk sugars.**Polysaccharides present in fungal/bacterial cell walls and biofilms, peptidoglycan, mutan, alginate, non-plant cell wall mannans.

†Polysaccharides present in fungal/bacterial cell walls and biofilms, e.g. peptidoglycan, mutan, alginate, non-plant cell wall mannans.

## Morphology

**Fig. 3.**
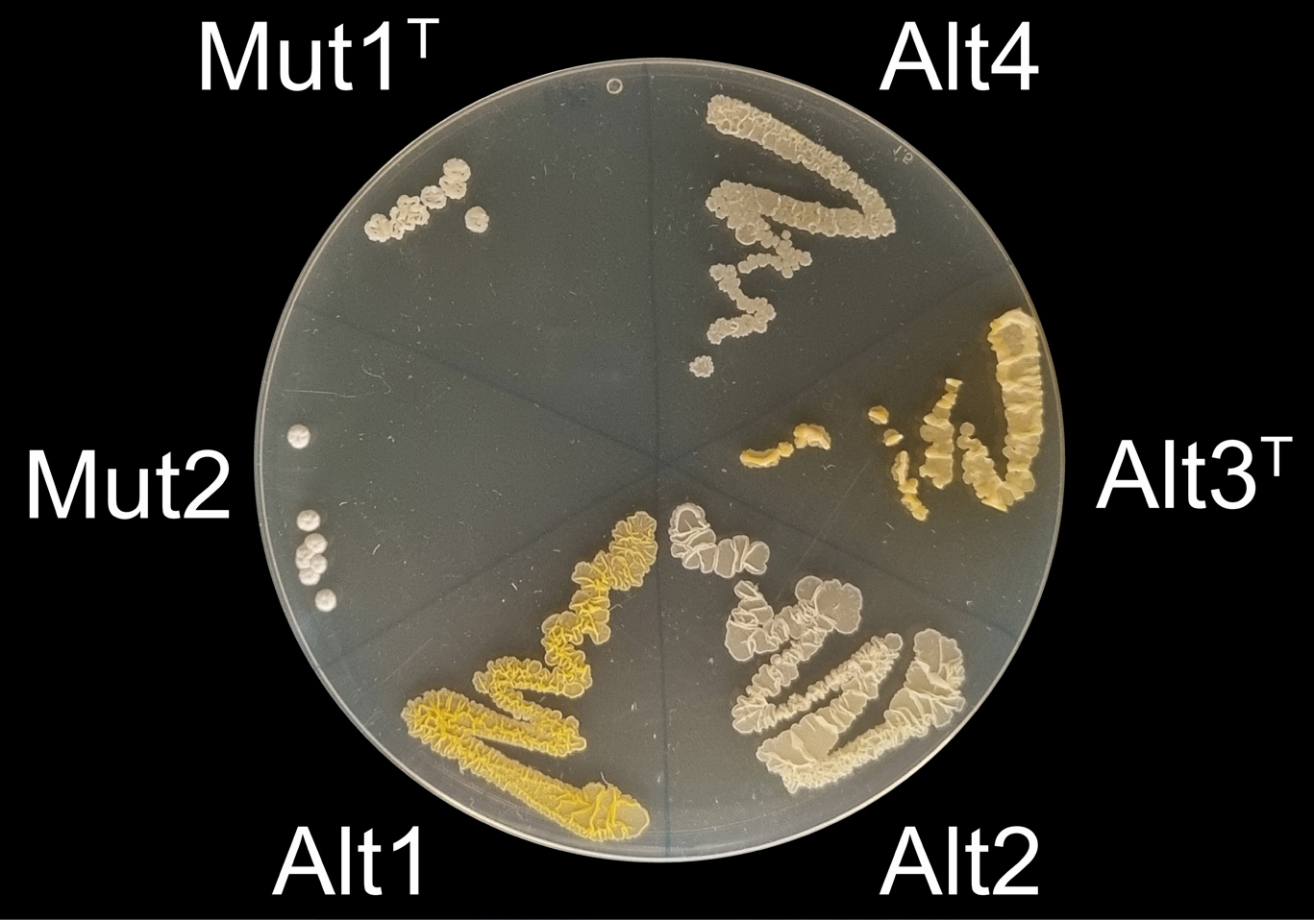
Growth of Mut1^T^, Mut2, Alt1, Alt2, Alt3^T^ and Alt4 on ISP2 agar after 7 days.

On ISP2 agar (yeast extract 4 g l^–1^, malt extract 10 g l^–1^, glucose 4 g l^–1^, agar 20 g l^–1^) [50], all six strains grew with similar morphologies, forming dry and hard/flaky colonies. However, the strains varied in pigmentation with Mut1^T^, Alt2, and Alt4 being off-white, Mut2 white, Alt1 strongly yellow and Alt3^T^ pale yellow, as can be seen in Fig. 3. After 7 days of growth on ISP2 medium, apparent spore formation was visible for Mut1^T^, Mut2 and Alt3^T^, based on a fluffy appearance, while similar features occurred later, after around 2 weeks of growth, for Alt1, Alt2 and Alt4. After 2 weeks of growth on ISP2 agar, Mut1^T^ and Alt1 showed secretion of yellow pigments (Fig. S5), Mut2 and Alt3^T^ showed weaker secretion of brown or yellow pigments, respectively, while Alt2 and Alt4 showed no secretion of pigments.

## Physiology

Optimal growth temperatures, pH, and salt (NaCl) tolerance were determined in liquid ISP2 media in triplicate cultivations over 4 days. For each experiment, the strains were precultured in ISP2 at 20 °C before inoculation with a starting optical density at 600 nm (OD_600_, measured in culture tubes with a CO7500 Colorimeter, WPA) of 0.01. The strains varied in morphology, with frequent formation of mycelial ball clusters of varying size as well as dispersed growth. The optimal growth temperature was determined at 8, 15, 20, 30, 37 and 42 °C, with cultures incubated with shaking at 250 r.p.m. and the OD_600_ of the cultures monitored. Mut1^T^ and Mut2 grew between 15 and 30 °C, while Alt1, Alt2 and Alt4 grew between 8 and 30 °C, and Alt3^T^ exhibited the narrowest temperature range and grew between 15 and 20 °C. All strains grew best at 20 °C based on observed biomass formation. The influence of pH for growth was determined between pH 4 and 10 with intervals of 1 pH unit. The pH value affected the ability of the strains to grow as well as their morphology (Fig. S6). All strains were able to grow between pH 5 and 10, and the apparent optimal growth pH was 5 for Mut1^T^ and Mut2, pH 6 for Alt1, Alt2 and Alt4, and pH 7 for Alt3^T^. It should be noted that all strains were able to acidify the media during growth (data not shown). Tolerance to NaCl was determined with salt concentrations of 0–10% with 1% intervals. Mut1^T^, Mut2, Alt1, Alt2 and Alt4 grew the best without any added salt while Alt3^T^ grew best at 3% NaCl. Mut1^T^ and Alt3^T^ grew in media with a NaCl concentration up to 6%, Mut2, Alt1 and Alt4 to 5% and Alt2 to 4%. For Alt1, Alt2 and Alt4, any addition of salt impacted the morphology substantially, causing formation of mycelial balls (Fig. S7).

**Fig. 4.**
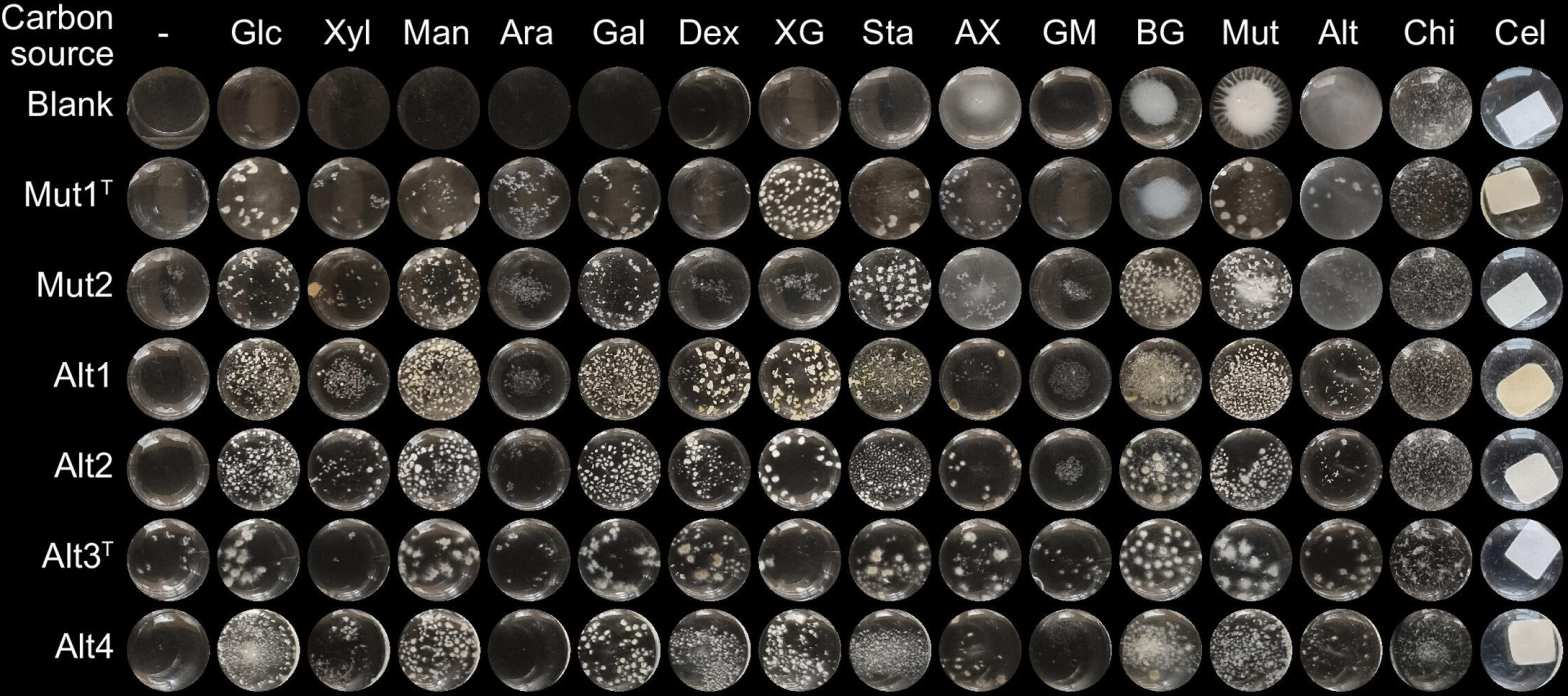
Growth of the isolates on different carbohydrates as carbon sources, from mono- to polysaccharides. Glc, d-glucose; Xyl, d-xylose; Man, d-mannose; Ara, l-arabinose; Gal, d-galactose; Dex, dextran; XG, xyloglucan; Sta, starch; AX, arabinoxylan; GM, galactomannan; BG, mixed-linkage β-glucan; Mut, mutan; Alt, alternan; Chi, chitin; Cel, cellulose. Note that some insoluble polysaccharides, such as mutan, appear as a white cluster at the bottom of the wells in the control samples, but this does not signify growth.

As mentioned previously, the strains were isolated on M9 medium including either mutan or alternan as sole carbon source, and growth on these polysaccharides was thus expected for the respective strains. In addition, based on the putative CAZymes present in their genomes, we expected growth to be possible on a wide range of additional polysaccharides. The growth of the isolates was therefore screened using M9 media supplemented with either the monosaccharides d-glucose, d-xylose, d-mannose, l-arabinose, or d-galactose, or the polysaccharides dextran, tamarind xyloglucan, starch, wheat arabinoxylan, carob galactomannan, yeast β-glucan (β−1,3-glucan with β−1,6 branches), mutan, alternan, shrimp shell chitin, or cellulose filter paper as sole carbon source. Strains Mut1^T^, Mut2, Alt1, Alt2 and Alt4 were precultured in ISP2, and Alt3^T^ in ISP2 supplemented with 3% NaCl. The cells were washed using centrifugation and inoculated to a final OD_600_ of 0.01 in 1 ml M9 media with 0.1% of respective carbon source in a 24-well plate. Blanks for all the carbon sources, and negative control cultures without a carbon source were included. The cultivations were performed in triplicate and the growth and ability to degrade substrates were assessed visually after 7 days, or 3 weeks for cellulose. Representative photos can be seen in Fig. 4 and interpretation of the growth in Table 5. All strains could grow on glucose, mannose, galactose, starch, mutan and cellulose. None could grow on arabinose and, unexpectedly, none were able to grow on galactomannan. Only Alt1, Alt2, Alt3^T^ and Alt4 could grow on dextran and alternan which might be explained by the presence of a predicted glycoside hydrolase family 49 (GH49) enzyme in these strains, which is an enzyme family known to contain dextranases. Surprisingly, only Alt3^T^ showed minimal, but clear, growth on chitin, despite the abundance of putative chitinases in the genomes of all strains. This might indicate that the enzymes target other β−1,4-*N*-acetylglucosamine-containing substrates such as *N*-glycans. Otherwise, the strains differed from each other by the following growth characteristics: Mut1^T^ could not grow on yeast β-glucan, Mut2 and Alt3^T^ could not grow on xyloglucan, Mut2 could not grow on arabinoxylan and Alt3^T^ could not grow on xylose. Possibly, the lack of growth could be explained by the absence of important degradative enzymes. Mut2 lacks enzymes from GH9 and GH12 that could potentially target the xyloglucan main chain, as well as GH11, GH30 and GH43 enzymes to target arabinoxylan. Evidently, the predicted activities from the encoded CAZymes could not always explain all growth behaviours of these strains. It should be noted that there are many forms of the same polysaccharide in nature, and that the ones tested in this study might not be the target of the present enzymes, or enzymes might only be expressed under specific conditions not tested here. The absence of growth should therefore not necessarily rule out the presence of any enzymes active on that polysaccharide. The growth on a substrate can, however, be taken as confirmation of the strains’ degradative abilities.

**Table 5.** Characteristics of srains Mut1^T^, Mut2, Alt1, Alt2, Alt3^T^ and Alt4, including growth substrates and enzymatic activities, compared with data for the most closely related type strains Mut1^T^ is most closely related to *Streptomyces drozdowiczii*, Mut2 to *Streptomyces laculatispora*, Alt1, Alt2 and Alt4 to *Streptomyces poriferorum*, and Alt3^T^ to *Streptomyces atroolivaceus*. +, Growth/enzymatic activity; −, no growth/no enzymatic activity; n.d.not determined; v, variable.

**Characteristics**	**Mut1** ^**T**^	**Mut2**	**Alt1**	**Alt2**	**Alt3** ^**T**^	**Alt4**	***S. drozdowiczii*** **NBRC 101007** ^**T**^ **[** **53** **]**	***S. laculatispora*** **BK166** ^**T**^ **[** **21** **]**	***S. poriferorum*** **P01-B04** ^**T**^ **[** **20** **]**	***S. atroolivaceus*** **NRRL ISP-5137** ^**T**^ **[** **54** **]**
Growth characteristics (ISP2 medium):										
Colour	Off-white	White	Yellow	Off-white	Pale yellow	Off-white	Grey-brown*	White	Pale yellow	Grey
Pigmentation	Yellow	Weak brown	Yellow	−	Weak yellow	−	Yellow-brown	−	n.d.	−
Temperature range (optimum) for growth (°C)	15–30 (20)	15–30 (20)	8–30 (20)	8–30 (20)	15–20 (20)	8–30 (20)	25–41(n.d.) *	4–30 (n.d.)	3–30 (25–30)	10–37 (n.d.)*
pH range (optimum) for growth	5–10 (5)	5–10 (5)	5–10 (6)	5–10 (6)	5–10 (7)	5–10 (6)	n.d.	5–9 (n.d.)	4.5–9.5 (7)	n.d.
NaCl range (optimum) for growth (%)	0–6 (0)	0–5 (0)	0–6 (0)	0–4 (0)	0–6 (3)	0–5 (0)	0–2 (n.d.)*	0–7 (n.d.)	0–5 (1–3)	0–6 (n.d.)*
Growth with single carbon sources (M9 medium):										
Glucose	+	+	+	+	+	+	n.d.	+	+	+
Xylose	+	+	+	+	−	+	−	+	+	+
Mannose	+	+	+	+	+	+	+	−	+	n.d.
Arabinose	−	−	−	−	−	−	+	+	+	+
Galactose	+	+	+	+	+	+	+	+	+	n.d.
Dextran	−	−	+	+	+	+	n.d.	n.d.	n.d.	n.d.
Xyloglucan	+	−	+	+	−	+	n.d.	n.d.	n.d.	n.d.
Starch	+	+	+	+	+	+	Degradation	Degradation	Degradation	n.d.
Arabinoxylan	+	−	+	+	+	+	Xylan −	Xylan +	n.d.	n.d.
Galactomannan	−	−	−	−	−	−	n.d.	n.d.	n.d.	n.d.
Yeast β-glucan	−	+	+	+	+	+	n.d.	n.d.	n.d.	n.d.
Mutan	+	+	+	+	+	+	n.d.	n.d.	n.d.	n.d.
Alternan	−	−	+	+	+	+	n.d.	n.d.	n.d.	n.d.
Chitin	−	−	−	−	+	−	n.d.	No degradation	n.d.	n.d.
Cellulose	+	+	+	+	+	+	Degradation	−	n.d.	n.d.
Enzymatic activities (API ZYM):										
Alkaline phosphatase	−	−	−	−	+	−	v*	n.d.	+	+*
Esterase (C4)	+	+	+	−	+	+	+*	n.d.	+	+*
Esterase lipase (C8)	+	+	+	+	+	+	+*	n.d.	+	+*
Lipase (C14)	−	−	−	−	−	−	v*	n.d.	−	+*
Leucine arylamidase	+	+	+	+	+	+	+*	n.d.	+	+*
Valine arylamidase	+	+	+	+	+	+	−*	n.d.	−	+*
Cystine arylamidase	−	−	−	−	−	−	−*	n.d.	−	+*
Trypsin	−	−	−	−	−	−	−*	n.d.	−	+*
α-Chymotrypsin	−	−	−	−	−	−	−*	n.d.	+	-*
Acid phosphatase	+	+	+	−	+	+	+*	n.d.	+	+*
Naphthol-AS-BI-phosphohydrolase	+	+	+	+	+	+	v*	n.d.	+	+*
α-Galactosidase	−	−	−	−	−	−	−*	n.d.	−	−*
β-Galactosidase	+	+	+	−	+	+	+*	n.d.	+	+*
β-Glucuronidase	−	−	−	−	−	−	−*	n.d.	−	−*
α-Glucosidase	+	+	−	−	+	−	v*	n.d.	+	+*
β-Glucosidase	+	+	+	+	+	+	+*	n.d.	+	+*
*N*-Acetyl-β-glucosaminidase	+	+	+	+	−	+	+*	n.d.	−	−*
α-Mannosidase	+	+	−	−	−	−	+*	n.d.	−	−*
α-Fucosidase	−	−	−	−	−	−	−*	n.d.	−	−*

*dData retrieved from the BacDive pages of *S. drozdowiczii* NBRC 101007T and *S. atroolivaceus* NRRL ISP-5137T (accessed 28 May 28, 2024) [55,57].

Additional enzymatic activities were determined using API ZYM (bioMérieux) according to the manufacturer’s instructions (Table 5), using cells grown in ISP2 medium (+3% NaCl for Alt3^T^). All strains showed activities of esterase lipase, leucine arylamidase, valine arylamidase, naphthol-AS-BI-phosphohydrolase, and β-glucosidase, but not of lipase, cystine arylamidase, trypsin, α-chymotrypsin, α-galactosidase, β-glucuronidase, or α-fucosidase. The strains had varying activities of alkaline phosphatase, esterase, acid phosphatase, β-galactosidase, α-glucosidase, *N*-acetyl-β-glucosaminidase, and α-mannosidase.

We then compared the phenotypic results of the isolated strains to the most closely related validly named species. Compared to *S. drozdowiczii* NBRC 101007^T^, Mut1^T^ displayed a narrower temperature growth range but ability to tolerate higher NaCl concentrations. The colour and the secretion of pigments is similar for both species. With regards to the carbon source utilization tested for both strains, Mut1^T^ cannot grow on arabinose but can grow on xylose and arabinoxylan (compared to an unspecified type of xylan *S. drozdowiczii* NBRC 101007^T^ could not utilize). API ZYM testing shows mostly similar results, but in contrast to *S. drozdowiczii* NBRC 101007^T^, Mut^T^ showed positive results for valine arylamidase. Compared to *S. laculatispora* BK166^T^ [21], Mut2 differed in its growth characteristics with an inability to grow at 8 °C and up to 7% NaCl. The carbon source utilization was also different, where Mut2, in contrast to *S. laculatispora*, could utilize mannose and cellulose, but not arabinose or arabinoxylan (compared to an unspecified type of xylan).

The identification of Alt1, Alt2 and Alt4 as strains of *S. poriferorum*, was supported by the growth characteristics with similar temperature, pH and, NaCl concentration growth ranges. However, in contrast to *S. poriferorum* P01-B04^T^, neither of Alt1, Alt2 or Alt4 could grow on arabinose as sole carbon source and the API ZYM test show positive results for valine arylamidase and negative for alkaline phosphatase, α-chymotrypsin and α-glucosidase. In addition, Alt2 differs from the other strains by being negative for esterase (C4), acid phosphatase, and β-galactosidase. Comparing Alt3^T^ with its most closely related type strain, *S. atroolivaceus* NRRL ISP-5137^T^, showed several differences in growth characteristics. Alt3^T^ is pale yellow and secretes yellow pigments on ISP2 agar while *S. atroolivaceus* NRRL ISP-5137^T^ is reported to be grey without any production of pigments. Alt3^T^ also has a narrower temperature growth range (15–20 °C) while *S. atroolivaceus* NRRL ISP-5137^T^ is reported to be able to grow at both lower and higher temperatures. The carbon source utilization also differs in that Alt3^T^ is unable to utilize xylose and arabinose, and with API ZYM, Alt3^T^ shows negative results for lipase (C14), cystine arylamidase, and trypsin.

Collectively, based on the phylogenetic and biochemical characterization we propose that Alt1, Alt2, and Alt4 are strains of *S. poriferorum,* Mut2 is a strain of *S. laculatispora,* and that Mut1^T^ and Alt3^T^ are recognized as the novel species *Streptomyces castrisilvae* sp. nov. and *Streptomyces glycanivorans* sp. nov. All strains were found to encode impressive arrays of CAZymes, and to have broad capabilities for degradation and metabolism of complex carbohydrates, and may be useful sources of clinically and industrially relevant enzymes.

## Description of *Streptomyces castrisilvae* sp. nov.

*Streptomyces castrisilvae* (cas.tri.sil’vae. L. neut. n. *castrum*, castle; L. fem. n. *silva*, forest; N.L. gen. n. *castrisilvae*, of castle forest, referring to the origin of the strain from soil in the park Slottsskogen (SWE: castle forest) in Gothenburg, Sweden).

Aerobic, filamentous *Actinomycetota* species. Grows as dry and flaky, off-white colonies on ISP2 agar with spore formation apparent after 1 week and secreted yellow pigments visible after 2 weeks. In liquid ISP2 it grows as mycelial balls under shaking conditions. Grows at temperatures between 15–30 °C, optimally at 20 °C, pH 5–10, and in the presence of 0–6% NaCl. Growth is observed in M9 minimal medium with glucose, xylose, mannose, galactose, xyloglucan, starch, arabinoxylan, mutan, and cellulose as sole carbon sources.

The type strain, Mut1^T^ (=DSM 117248^T^=CCUG 77596^T^), was isolated from a soil sample from the park Slottsskogen in Gothenburg, Sweden. The genome size is 7 990 897 bp with a G+C content of 72%. The GenBank genome accession number is CP120997.1 and the 16S rRNA gene sequence accession number is PP532741.

## Description of *Streptomyces glycanivorans* sp. nov.

*Streptomyces glycanivorans* (gly.ca.ni.vo’rans. N.L. neut. n. *glycanum*, glycan, a heteropolysaccharide; L. pres. part. *vorans*, eating, devouring; N.L. part. adj. *glycanivorans*, glycan devouring, referring to the ability of the strain to utilize multiple complex polysaccharides (glycans) as carbon source).

Aerobic, filamentous *Actinomycetota* species. Grows as dry and flaky, pale-yellow colonies on ISP2 agar with spore formation apparent after 1 week and secreted yellow pigments visible after 2 weeks. In liquid ISP2 it grows as mycelial balls under shaking conditions. Grows at 15–20 °C (optimally at 20 °C), pH 5–10, and in the presence of 0–6 % NaCl. Growth is observed in M9 minimal medium with glucose, mannose, galactose, dextran, starch, arabinoxylan, yeast β-glucan, mutan, alternan, chitin, and cellulose as sole carbon sources.

The type strain, Alt3^T^ (=DSM 117252^T^=CCUG 77600^T^), was isolated from a soil sample from the park Kungsparken in Gothenburg, Sweden. The genome size is 8 654 387 bp with a G+C content of 70.5%. The GenBank genome accession number is CP120983.1 and the 16S rRNA gene sequence accession number is PP532745.

## supplementary material

10.1099/ijsem.0.006514 Uncited Supplementary Material 1.

## Abbreviations

ANI: average nucleotide identity
CAZymes: carbohydrate-active enzymes
dDDH: digital DNA–DNA hybridization
GH: glycoside hydrolase family
ISP: International *Streptomyces* Project
OGRI: overall genomic relatedness index

## References

[1] Parte AC, Sardà Carbasse J, Meier-Kolthoff JP, Reimer LC, Göker M (2020). List of Prokaryotic names with Standing in Nomenclature (LPSN) moves to the DSMZ. Int J Syst Evol Microbiol.

[2] Nikolaidis M, Hesketh A, Frangou N, Mossialos D, Van de Peer Y (2023). A panoramic view of the genomic landscape of the genus *Streptomyces*. Microb Genom.

[3] Chater KF, Biró S, Lee KJ, Palmer T, Schrempf H (2010). The complex extracellular biology of *Streptomyces*. FEMS Microbiol Rev.

[4] Kieser T, Bibb MJ, Buttner MJ, Chater KF, Hopwood DA (2000). Practical Streptomyces Genetics.

[5] Chater KF (2016). Recent advances in understanding *Streptomyces*. F1000Res.

[6] Spasic J, Mandic M, Djokic L, Nikodinovic-Runic J (2018). *Streptomyces* spp. in the biocatalysis toolbox. Appl Microbiol Biotechnol.

[7] Pradeep GC, Choi YH, Choi YS, Suh SE, Seong JH (2014). An extremely alkaline novel chitinase from *Streptomyces* sp. CS495. Process Biochem.

[8] Beg QK, Bhushan B, Kapoor M, Hoondal GS (2000). Production and characterization of thermostable xylanase and pectinase from *Streptomyces* sp. QG-11-3. J Ind Microbiol Biotechnol.

[9] Grigorevski de Lima AL, Pires do Nascimento R, da Silva Bon EP, Coelho RRR (2005). *Streptomyces drozdowiczii* cellulase production using agro-industrial by-products and its potential use in the detergent and textile industries. Enzyme Microb Technol.

[10] Drula E, Garron M-L, Dogan S, Lombard V, Henrissat B (2022). The carbohydrate-active enzyme database: functions and literature. Nucleic Acids Res.

[11] Simpson CL, Cheetham NWH, Jacques NA (1995). Four glucosyltransferases, GtfJ, GtfK, GtfL and GtfM, from *Streptococcus salivarius* ATCC 25975. Microbiology.

[12] Koo H, Falsetta ML, Klein MI (2013). The exopolysaccharide matrix: a virulence determinant of cariogenic biofilm. J Dent Res.

[13] Fuglsang CC, Berka RM, Wahleithner JA, Kauppinen S, Shuster JR (2000). Biochemical analysis of recombinant fungal mutanases. A new family of alpha1,3-glucanases with novel carbohydrate-binding domains. J Biol Chem.

[14] Yano S, Wakayama M, Tachiki T (2006). Cloning and expression of an alpha-1,3-glucanase gene from *Bacillus circulans* KA-304: the enzyme participates in protoplast formation of *Schizophyllum commune*. Biosci Biotechnol Biochem.

[15] Suyotha W, Yano S, Itoh T, Fujimoto H, Hibi T (2014). Characterization of α-1,3-glucanase isozyme from *Paenibacillus glycanilyticus* FH11 in a new subgroup of family 87 α-1,3-glucanase. J Biosci Bioeng.

[16] Itoh T, Panti N, Hayashi J, Toyotake Y, Matsui D (2020). Crystal structure of the catalytic unit of thermostable GH87 α-1,3-glucanase from *Streptomyces thermodiastaticus* strain HF3-3. Biochem Biophys Res Commun.

[17] Okazaki K, Amano T, Morimoto T, Iemoto T, Kawabata T (2001). Cloning and nucleotide sequence of the mycodextranase gene from *Streptomyces* sp. J-13-3. Biosci Biotechnol Biochem.

[18] Leathers TD, Nunnally MS, Côté GL (2009). Modification of alternan by dextranase. Biotechnol Lett.

[19] Park T-S, Jeong HJ, Ko J-A, Ryu YB, Park S-J (2012). Biochemical characterization of thermophilic dextranase from a thermophilic bacterium, *Thermoanaerobacter pseudethanolicus*. J Microbiol Biotechnol.

[20] Sandoval-Powers M, Králová S, Nguyen G-S, Fawwal DV, Degnes K (2021). *Streptomyces poriferorum* sp. nov., a novel marine sponge-derived *Actinobacteria* species expressing anti-MRSA activity. *Syst Appl Microbiol*.

[21] Zucchi TD, Kim B-Y, Kshetrimayum JD, Weon H-Y, Kwon S-W (2012). *Streptomyces brevispora* sp. nov. and *Streptomyces laculatispora* sp. nov., actinomycetes isolated from soil. Int J Syst Evol Microbiol.

[22] Puanglek S, Kimura S, Enomoto-Rogers Y, Kabe T, Yoshida M (2016). *In vitro* synthesis of linear α-1,3-glucan and chemical modification to ester derivatives exhibiting outstanding thermal properties. Sci Rep.

[23] Altschul SF, Gish W, Miller W, Myers EW, Lipman DJ (1990). Basic local alignment search tool. J Mol Biol.

[24] De Coster W, D’Hert S, Schultz DT, Cruts M, Van Broeckhoven C (2018). NanoPack: visualizing and processing long-read sequencing data. Bioinformatics.

[25] Lin Y, Yuan J, Kolmogorov M, Shen MW, Chaisson M (2016). Assembly of long error-prone reads using de Bruijn graphs. Proc Natl Acad Sci USA.

[26] Wick RR, Schultz MB, Zobel J, Holt KE (2015). Bandage: interactive visualization of *de novo* genome assemblies. Bioinformatics.

[27] Parks DH, Imelfort M, Skennerton CT, Hugenholtz P, Tyson GW (2015). CheckM: assessing the quality of microbial genomes recovered from isolates, single cells, and metagenomes. Genome Res.

[28] Chaumeil P-A, Mussig AJ, Hugenholtz P, Parks DH (2020). GTDB-Tk: a toolkit to classify genomes with the Genome Taxonomy Database. Bioinformatics.

[29] Parks DH, Chuvochina M, Chaumeil P-A, Rinke C, Mussig AJ (2020). A complete domain-to-species taxonomy for bacteria and archaea. Nat Biotechnol.

[30] Schwengers O, Jelonek L, Dieckmann M, Beyvers S, Blom J (2021). Bakta: rapid & standardized annotation of bacterial genomes via alignment-free sequence identification. Bioinformatics.

[31] Lipun K, Chantavorakit T, Mingma R, Duangmal K (2020). *Streptomyces acidicola* sp. nov., isolated from a peat swamp forest in Thailand. J Antibiot.

[32] Caicedo-Montoya C, Manzo-Ruiz M, Ríos-Estepa R (2021). Pan-genome of the genus *Streptomyces* and prioritization of biosynthetic gene clusters with potential to produce antibiotic compounds. Front Microbiol.

[33] Yoon S-H, Ha S-M, Kwon S, Lim J, Kim Y (2017). Introducing EzBioCloud: a taxonomically united database of 16S rRNA gene sequences and whole-genome assemblies. Int J Syst Evol Microbiol.

[34] Saitou N, Nei M (1987). The neighbor-joining method: a new method for reconstructing phylogenetic trees. Mol Biol Evol.

[35] Tamura K, Stecher G, Kumar S (2021). MEGA11: Molecular Evolutionary Genetics Analysis version 11. Mol Biol Evol.

[36] Madhaiyan M, Saravanan VS, See-Too W-S, Volpiano CG, Sant’Anna FH (2022). Genomic and phylogenomic insights into the family *Streptomycetaceae* lead to the proposal of six novel genera. Int J Syst Evol Microbiol.

[37] Felsenstein J (1985). Confidence limits on phylogenies: an approach using the bootstrap. Evolution.

[38] Tamura K, Nei M, Kumar S (2004). Prospects for inferring very large phylogenies by using the neighbor-joining method. Proc Natl Acad Sci USA.

[39] Meier-Kolthoff JP, Göker M (2019). TYGS is an automated high-throughput platform for state-of-the-art genome-based taxonomy. Nat Commun.

[40] Meier-Kolthoff JP, Auch AF, Klenk H-P, Göker M (2013). Genome sequence-based species delimitation with confidence intervals and improved distance functions. BMC Bioinform.

[41] Riesco R, Trujillo ME (2024). Update on the proposed minimal standards for the use of genome data for the taxonomy of prokaryotes. Int J Syst Evol Microbiol.

[42] Komaki H (2022). Resolution of housekeeping gene sequences used in MLSA for the genus *Streptomyces* and reclassification of *Streptomyces anthocyanicus* and *Streptomyces tricolor* as heterotypic synonyms of *Streptomyces violaceoruber*. Int J Syst Evol Microbiol.

[43] Labeda DP, Dunlap CA, Rong X, Huang Y, Doroghazi JR (2017). Phylogenetic relationships in the family *Streptomycetaceae* using multi-locus sequence analysis. Antonie van Leeuwenhoek.

[44] Meier-Kolthoff JP, Carbasse JS, Peinado-Olarte RL, Göker M (2022). TYGS and LPSN: a database tandem for fast and reliable genome-based classification and nomenclature of prokaryotes. Nucleic Acids Res.

[45] Richter M, Rosselló-Móra R, Oliver Glöckner F, Peplies J (2016). JSpeciesWS: a web server for prokaryotic species circumscription based on pairwise genome comparison. Bioinformatics.

[46] Hu S, Li K, Zhang Y, Wang Y, Fu L (2022). New insights into the threshold values of multi-locus sequence analysis, average nucleotide identity and digital DNA-DNA hybridization in delineating *Streptomyces* species. Front Microbiol.

[47] Chevrette MG, Carlson CM, Ortega HE, Thomas C, Ananiev GE (2019). The antimicrobial potential of *Streptomyces* from insect microbiomes. Nat Commun.

[48] Andam CP, Doroghazi JR, Campbell AN, Kelly PJ, Choudoir MJ (2016). A latitudinal diversity gradient in terrestrial bacteria of the genus *Streptomyces*. mBio.

[49] Blin K, Shaw S, Augustijn HE, Reitz ZL, Biermann F (2023). antiSMASH 7.0: new and improved predictions for detection, regulation, chemical structures and visualisation. Nucleic Acids Res.

[50] Shirling EB, Gottlieb D (1966). Methods for characterization of *Streptomyces* species. Int J Syst Bacteriol.

[51] Kitts PA, Church DM, Thibaud-Nissen F, Choi J, Hem V (2016). Assembly: a resource for assembled genomes at NCBI. Nucleic Acids Res.

[52] Lefort V, Desper R, Gascuel O (2015). FastME 2.0: a comprehensive, accurate, and fast distance-based phylogeny inference program. Mol Biol Evol.

[53] Semêdo LTAS, Gomes RC, Linhares AA, Duarte GF, Nascimento RP (2004). *Streptomyces drozdowiczii* sp. nov., a novel cellulolytic streptomycete from soil in Brazil. Int J Syst Evol Microbiol.

[54] Shirling EB, Gottlieb D (1968). Cooperative description of type cultures of *Streptomyces*.: II. Species descriptions from first study. Int J Syst Bacteriol.

[55] Reimer LC, Sarda Carbasse J, Koblitz J, Podstawka A, Overmann J *Streptomyces drozdowiczii* (Semêdo *et al*. 2004).

[56] Reimer LC, Sarda Carbasse J, Koblitz J, Podstawka A, Overmann J *Streptomyces atroolivaceus* (Preobrazhenskaya *et al*. 1957) Pridham *et al*. 1958 emend. Nouioui *et al*. 2018.

[57] Reimer LC, Sardà Carbasse J, Koblitz J, Ebeling C, Podstawka A (2022). BacDive in 2022: the knowledge base for standardized bacterial and archaeal data. Nucleic Acids Res.

