# Supplementary material for: Streptomyces castrisilvae sp. nov. and Streptomyces glycanivorans sp. nov., novel soil streptomycetes metabolizing mutan and alternan
Source: Int J Syst Evol Microbiol. 2024 Sep 12;74(9):006514. doi: 10.1099/ijsem.0.006514 (PMC11475409; doi:10.1099/ijsem.0.006514)
Supplement: Uncited Supplementary Material 1. [file ijsem-74-06514-s001.pdf]

Supplementary Information for:

Streptomyces castrisilvae sp. nov. and *Streptomyces glycanivorans* sp. nov., novel soil streptomycetes metabolizing mutan and alternan

Tove Widén[1], Albert Tafur Rangel[1,2,#], Vincent Lombard[3], Elodie Drula[3], Scott Mazurkewich[1], Nicolas Terrapon[3], Eduard J. Kerkhoven[1,2], Johan Larsbrink[1,4,*]

Affiliations:

[1] Department of Life Sciences, Chalmers University of Technology, SE-412 96 Gothenburg, Sweden

[2] Novo Nordisk Foundation Center for Biosustainability, Technical University of Denmark, DK-2800 Kgs. Lyngby, Denmark

[3] Architecture et Fonction des Macromolécules Biologiques, USC 1408 INRAE, UMR 7257 AMU, CNRS, FR-13288 Marseille, France

[4] Wallenberg Wood Science Center, Chalmers University of Technology, SE-412 96, Gothenburg, Sweden

* correspondence: Johan Larsbrink, johan.larsbrink@chalmers.se

[#] present address: Department of Microbiology, Universidad Popular del Cesar, Valledupar, Colombia

Supplementary Tables

Table S1. Antismash results for Mut1^{T}. NRP = nonribosomal peptide, RiPP = ribosomally synthesized and post-translationally modified peptide.

Region	Type	From	To	Most similar known cluster and metabolite type		Similarity to known gene clusters
Region 1	NRPS, betalactone	70 774	113 168			
Region 2	NRPS	137 692	225 650	cadaside A/cadaside B	NRP	19%
Region 3	T2PKS, terpene	226 925	299 428	spore pigment	Polyketide	83%
Region 4	T3PKS, NRPS, NRP-metallophore, betalactone	363 786	450 750	coelichelin	NRP	100%
Region 5	NRPS-like, NRPS	461 304	542 236	capreomycin IA/capreomycin IB/capreomycin IIA/capreomycin IIB	NRP	12%
Region 6	NRP-metallophore, NRPS	618 806	667 209	paenibactin	NRP	83%
Region 7	T1PKS	982 847	1 026 520	sporolide A/sporolide B	NRP+Polyketide: Enediyne type I polyketide	25%
Region 8	terpene	1 041 275	1 060 566	steffimycin D	Polyketide: Type II polyketide + Saccharide: Hybrid/tailoring saccharide	19%
Region 9	phosphonate, RRE-containing	1 170 986	1 201 427	dehydrofosmidomycin	Other	23%
Region 10	ectoine	1 532 279	1 542 677	ectoine	Other	100%
Region 11	indole	2 505 503	2 526 759	5-isoprenylindole-3-carboxylate β-D-glycosyl ester	Other	28%
Region 12	LAP, thiopeptide	2 662 562	2 696 029			
Region 13	lanthipeptide-class-ii	2 930 600	2 958 685	birimositide	RiPP: lanthipeptide	75%
Region 14	T1PKS, NRPS-like, transAT-PKS-like	3 270 060	3 319 364	cinnabaramide A	NRP+Polyketide: Modular type I polyketide	18%

Region 15	T1PKS, butyrolactone	3 927 532	4 042 561	bombyxamycin A/bombyxamycin B	Polyketide	28%
Region 16	LAP	4 515 691	4 535 454			
Region 17	lassopeptide	4 594 231	4 616 639	pentamycin	Polyketide	20%
Region 18	NI-siderophore	5 963 512	5 978 083	schizokinen	Other	25%
Region 19	thiopeptide, LAP	6 109 702	6 138 626	bombyxamycin A/bombyxamycin B	Polyketide	9%
Region 20	RiPP-like	6 316 394	6 327 812			
Region 21	NRPS	6 605 703	6 673 305	bosamycin A/bosamycin B/bosamycin C/bosamycin D/bosamycin E/bosamycin F	NRP	33%
Region 22	terpene	7 048 522	7 075 130	hopene	Terpene	76%
Region 23	T3PKS	7 515 315	7 556 364	violapyrone B	Polyketide	28%
Region 24	hglE-KS, lanthipeptide-class-iv	7 569 628	7 622 559	venezuelin	RiPP: Lanthipeptide	100%

Table S2. Antismash results for Alt3^T. NRP = nonribosomal peptide, RiPP = ribosomally synthesized and post-translationally modified peptide.

**Region**	**Type**	**From**	**To**	**Most similar known cluster and metabolite type**		**Similarity to known gene clusters**
Region 1	T1PKS	43 944	91 850	SGR PTMs/SGR PTM Compound b/ SGR PTM Compound c/SGR PTM Compound d	NRP + Polyketide	100%
Region 2	NRP-metallophore, NRPS	271 760	329 632	coelichelin	NRP	90%
Region 3	terpene	357 460	381 334	isorenieratene	Terpene	100%
Region 4	RiPP-like	539 392	548 871			
Region 5	NRP-metallophore, NRPS	583 333	631 858	griseobactin	NRP	61%
Region 6	blactam	636 515	660 013	clavulanic acid	Other: Non-NRP beta-lactam	20%
Region 7	terpene	736 147	761 964	hopene	Terpene	76%

Region 8	T1PKS	1 216 199	1 310 868	tripartilactam/niizalactam C	Polyketide	96%
Region 9	RiPP-like	1 540 743	1 550 746			
Region 10	ladderane	1 690 834	1 732 021			
Region 11	NRPS	1 776 947	1 838 544	detoxin S1	NRP + Polyketide	66%
Region 12	NI-siderophore	2 175 186	2 188 462	schizokinen	Other	25%
Region 13	terpene	2 254 040	2 274 180			
Region 14	RiPP-like	2 435 178	2 443 194			
Region 15	butyrolactone	4 262 006	4 271 521	zorbamycin	NRP: Glycopeptide + Polyketide: Modular type I polyketide + Saccharide: Hybrid/tailoring saccharide	4%
Region 16	NRPS-like, T1PKS, NRPS	4 497 851	4 551 984	istamycin	Saccharide	11%
Region 17	NI-siderophore	5 244 323	5 256 104	desferrioxamin B	Other	100%
Region 18	lanthipeptide-class-iii, lanthipeptide-class-ii	5 319 032	5 349 985			
Region 19	lanthipeptide-class-i	5 669 815	5 694 163			
Region 20	NRPS	5 763 131	5 817 002	enduracididine	Other	20%
Region 21	terpene	5 954 125	5 974 532			
Region 22	betalactone, butyrolactone	6 399 066	6 431 439	A-201A	Other	6%
Region 23	ectoine	6 455 876	6 464 980	ectoine	Other	100%
Region 24	lanthipeptide-class-i	6 807 820	6 833 082			
Region 25	T2PKS, PKS-like	6 954 539	7 026 104	streptoketide A/streptoketide B/streptoketide C/S2502/S2507/UWM5	Polyketide	100%
Region 26	terpene	7 071 596	7 091 395	steffimycin D	Polyketide: Type II polyketide + Saccharide: Hybrid/tailoring saccharide	16%
Region 27	terpene, ectoine, hglE-KS, T1PKS	7 405 774	7 480 559	ectoine	Other	100%
Region 28	RiPP-like	7 499 703	7 509 930	hexacosalactone A	Other	4%

Region 29	T2PKS	7 604 460	7 676 094	saccharothrixin D/saccharothrixin E/saccharothrixin F/ saccharothrixin G/saccharothrixin H/saccharothrixin I/ saccharothrixin J/saccharothrixin K/saccharothrixin L/saccharothrixin M	Polyketide	55%
Region 30	T3PKS	7 717 614	7 758 672	tetronasin	Polyketide	11%
Region 31	melanin	7 848 533	7 858 997	melanin	Other	100%
Region 32	T2PKS, terpene	7 901 776	7 974 303	spore pigment	Polyketide	83%
Region 33	NRPS	8 179 526	8 273 884	detoxin S1	NRP + Polyketide	33%

Supplementary Figures

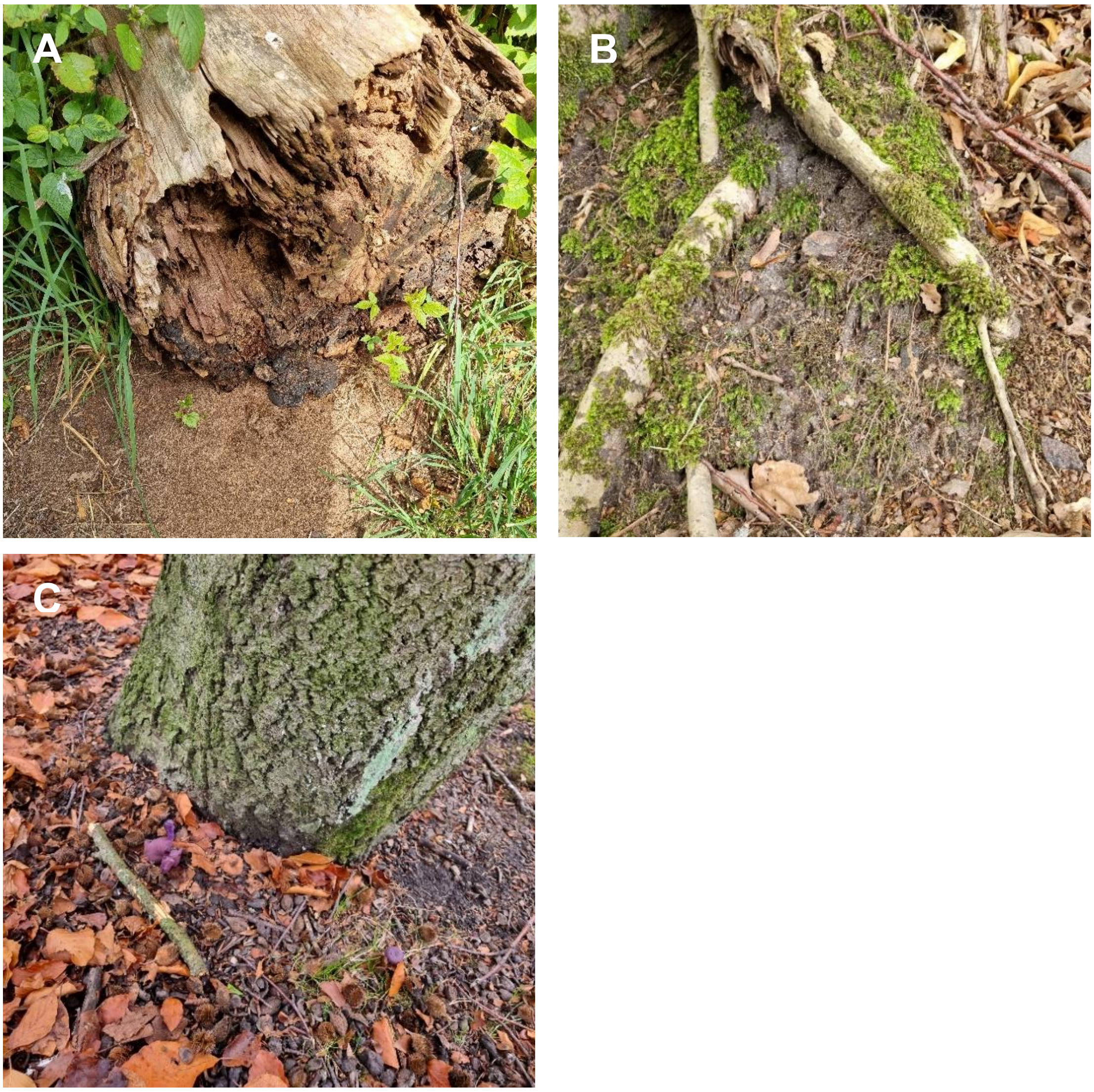

Figure S1. Images of the locations from where the soil samples for the strain isolations were taken. A) Sample collection site for strains Mut1^{T}, B) sample collection site for strain Mut2, and C) sample collection site for strains Alt1, Alt2, Alt3^{T} and Alt4.

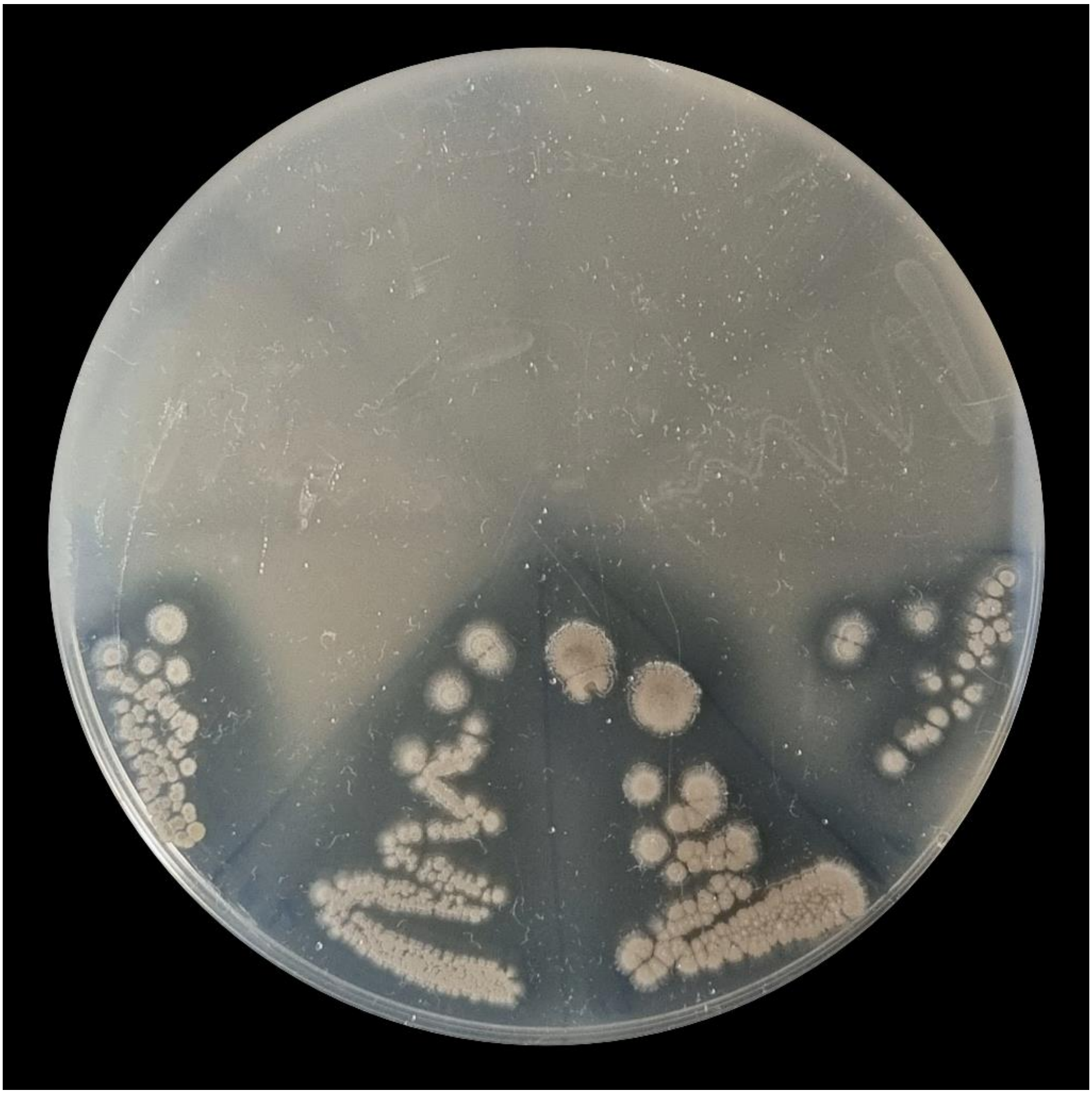

Figure S2. Example of clearing zones around bacteria degrading alternan on an agar plate.

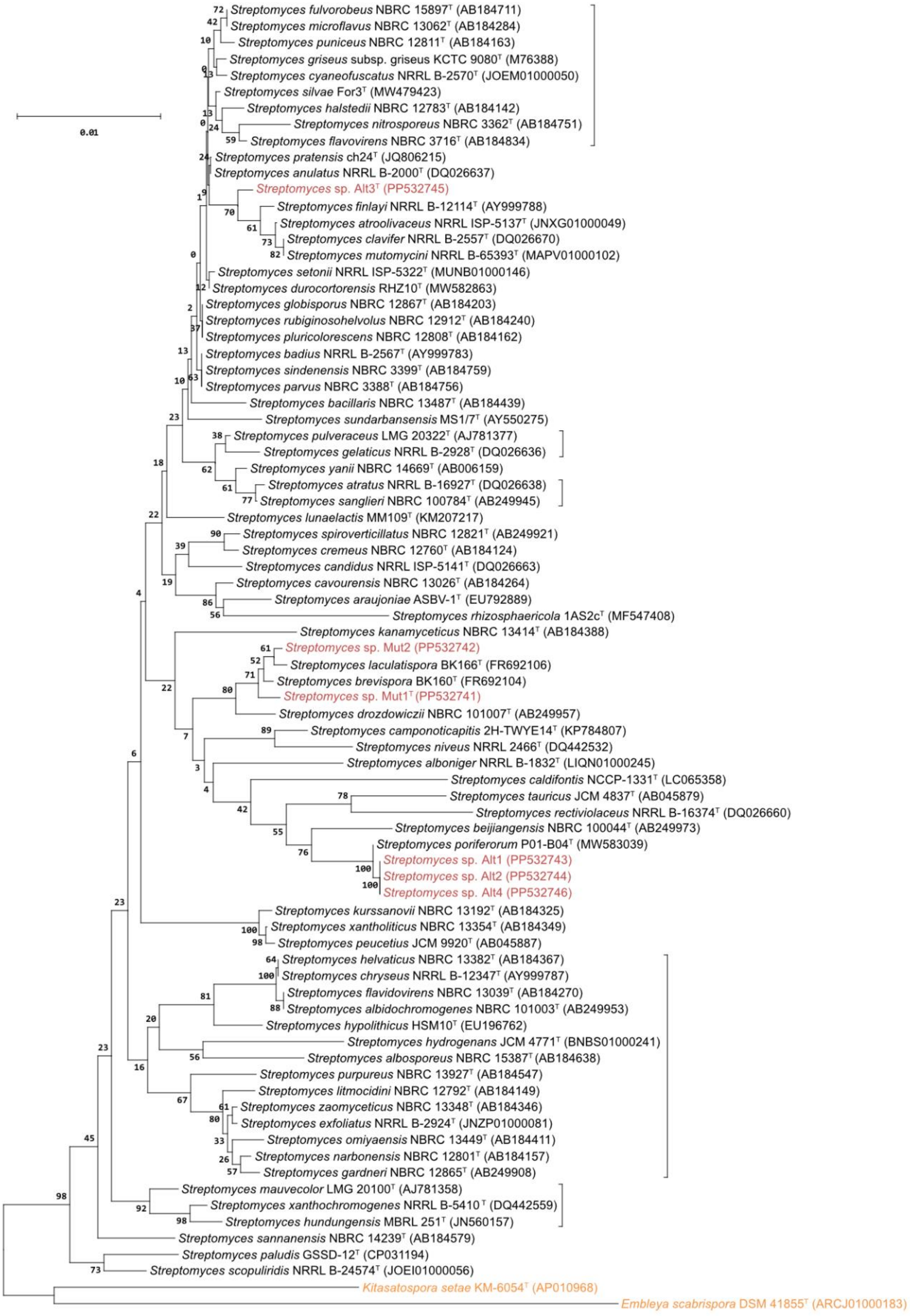

Figure S3. Full phylogenetic tree of *Streptomyces* sp. Alt1, Alt2, Alt3, Alt4, Mut1, Mut2 from 16S rRNA gene sequences. The tree was reconstructed using the neighbor-joining method. The root position of the neighbor-joining tree was determined using *Kitasatospora setae* KM-6054^{T} (AP010968) and *Embleya scabrispora* DSM 41855^{T} (ARCJ01000183) as the outgroup. Bold numbers on the branches are the confidence limits estimated by bootstrap analysis with 1000 replicates. Collapsed nodes on Figure 1 are shown within brackets. The strains investigated in this study are shown in red, based on their grouping. Strains in orange are outgroups.

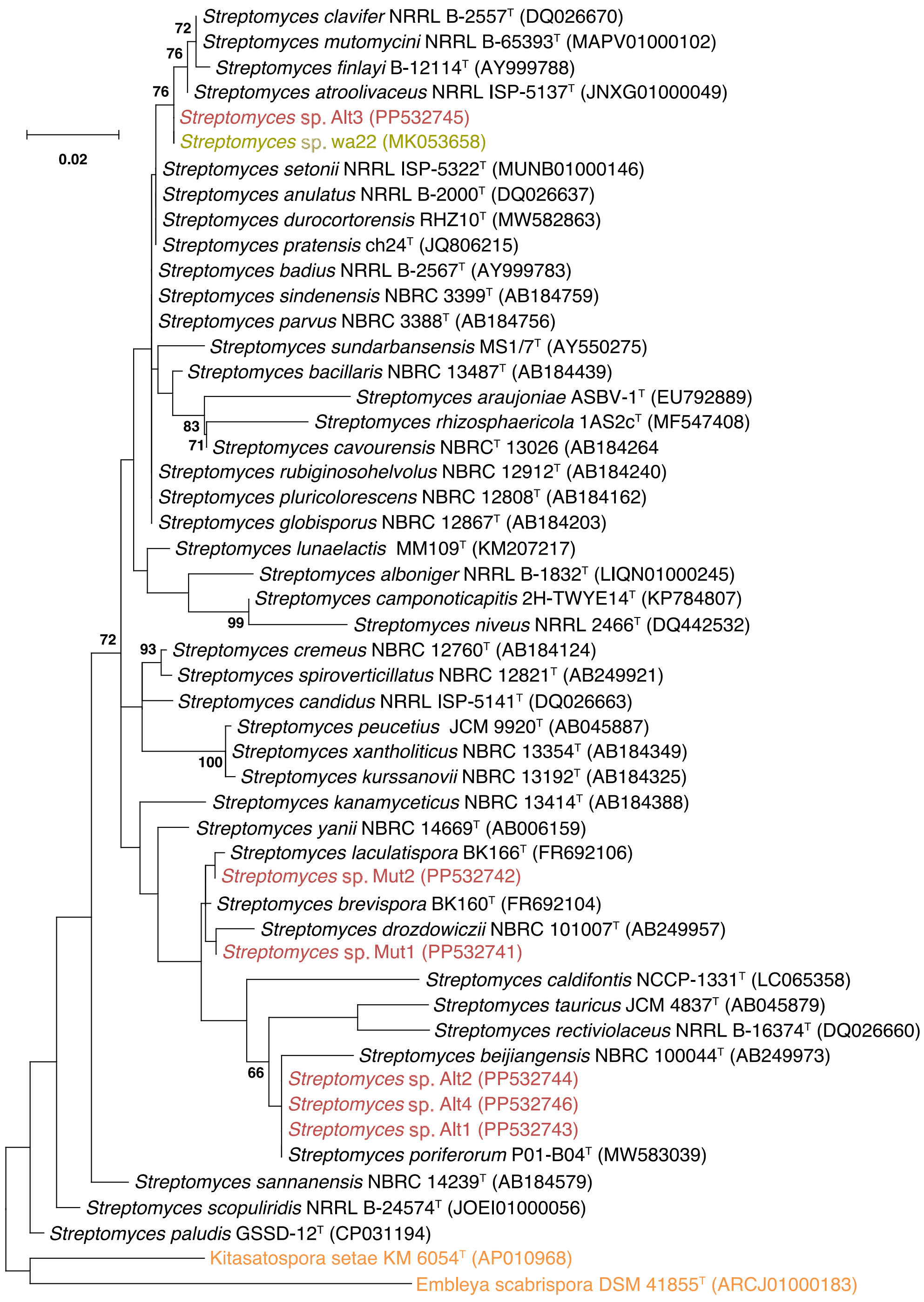

Figure S4. Maximum Likelihood (ML) tree inferred by the GTR+CAT model and rooted by outgroup. The branches are scaled in terms of the expected number of substitutions per site. The numbers above the branches are support values when larger than 60% from ML and Maximum Parsimony (MP) bootstrapping. Pairwise sequence similarities were calculated using the method recommended by Meier-Kolthoff et al. [1] for the 16S rRNA gene sequences available via the GGDC web server [2], at http://ggdc.dsmz.de/. Phylogenies were inferred by the GGDC web server [2], available at http://ggdc.dsmz.de/, using the DSMZ phylogenomics pipeline [3] adapted to single genes. A multiple sequence alignment was created with MUSCLE [4]. ML and MP trees were inferred from the alignment with RAxML [5] and TNT [6], respectively. For ML, rapid bootstrapping in conjunction with the autoMRE bootstopping criterion [7] and subsequent search for the best tree was used; for MP, 1000 bootstrapping replicates were used in conjunction with tree-bisection-and-reconnection branch swapping and ten random sequence addition replicates. The sequences were checked for a compositional bias using the X^2 test as implemented in PAUP* [8].

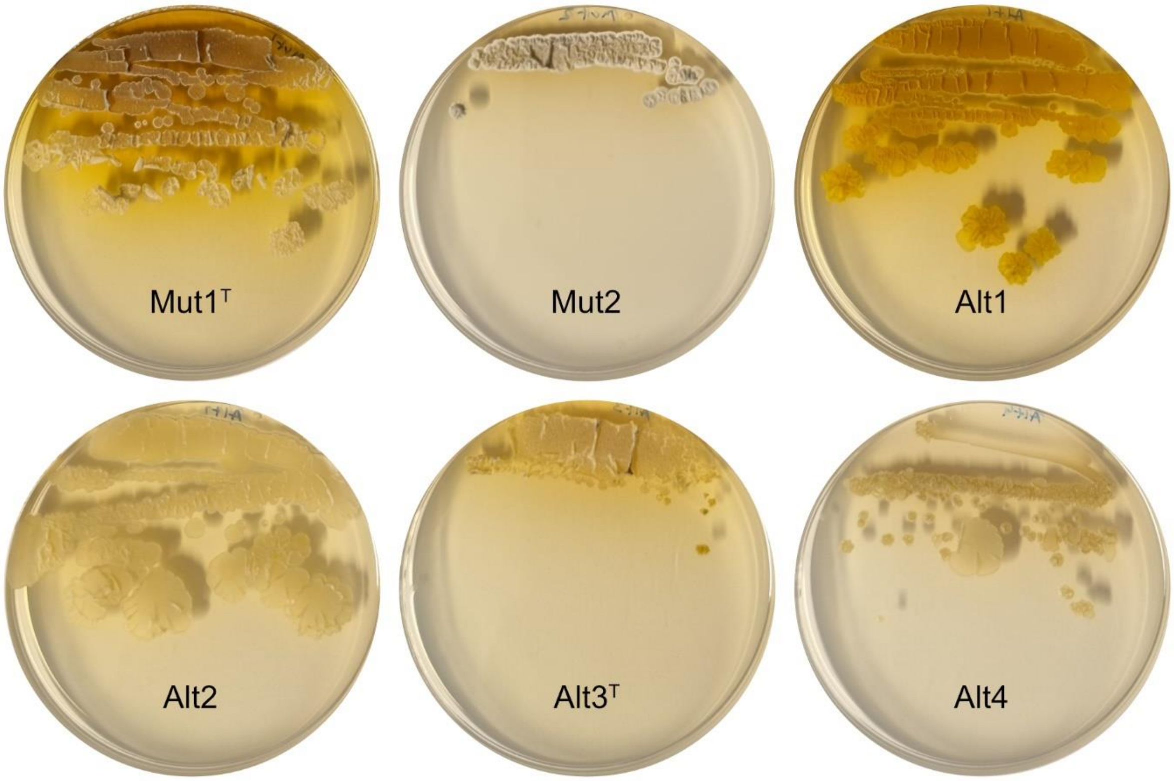

Figure S5. Growth of Mut1^T, Mut2, Alt1, Alt2, Alt3^T and Alt4 on ISP2 agar after two weeks.

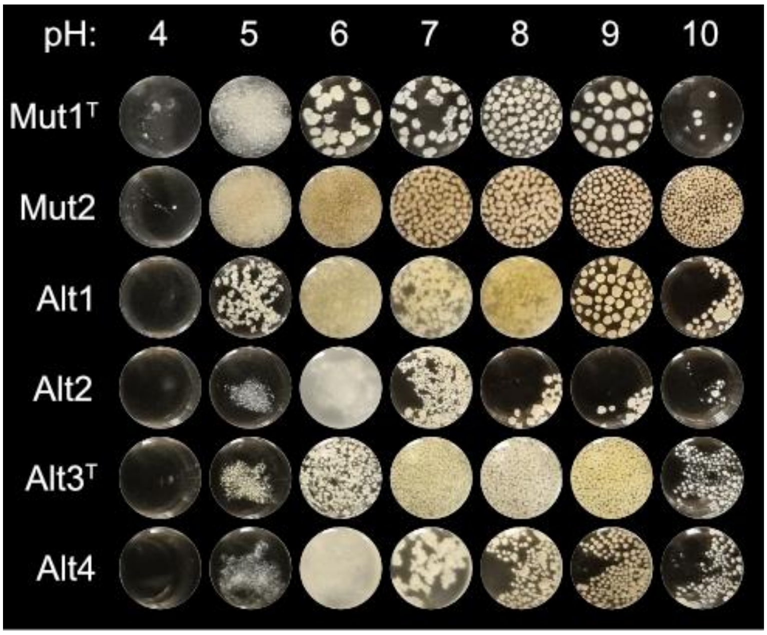

Figure S6. Growth of the isolates in liquid ISP2 medium at different pH values after four days. At different pH values, the growth was either dispersed or led to formation of mycelial balls.

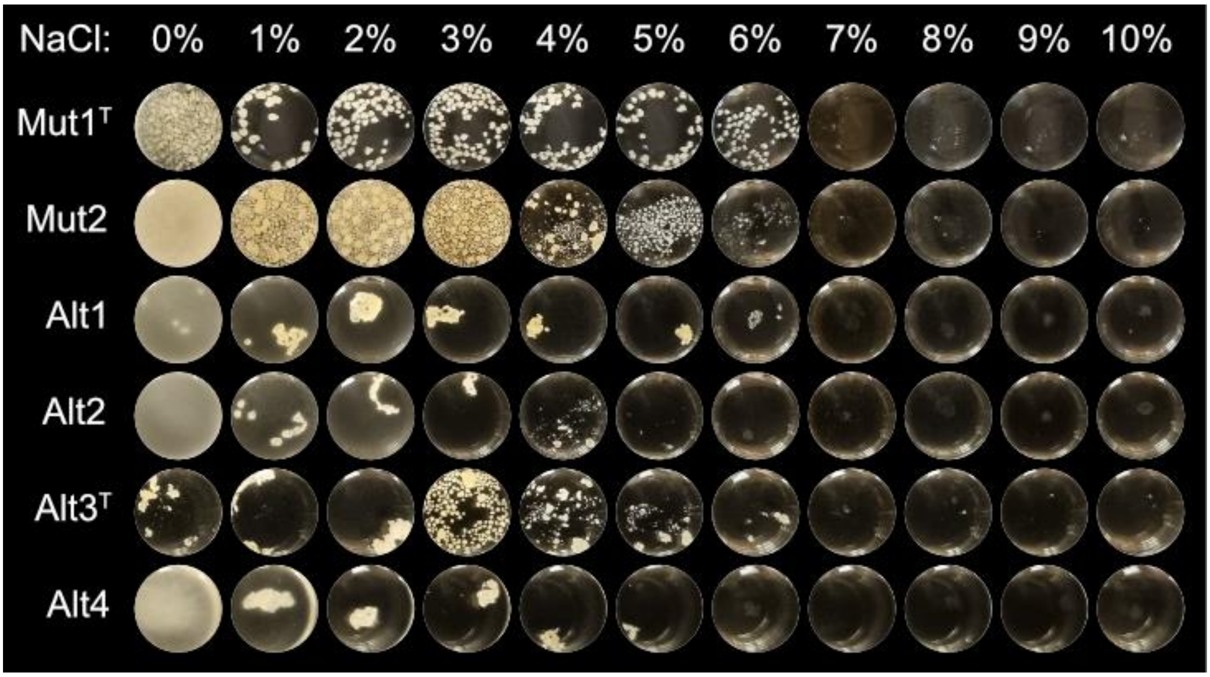

Figure S7. Growth of the isolates in liquid ISP2 medium with different concentrations of NaCl after four days.

<u>**Supplementary references**</u>

1. **Meier-Kolthoff JP, Göker M, Spröer C, Klenk H-P**. When should a DDH experiment be mandatory in microbial taxonomy? *Arch Microbiol* 2013;195:413–418:DOI: 10.1007/s00203-013-0888-4.

2. **Meier-Kolthoff JP, Carbasse JS, Peinado-Olarte RL, Göker M**. TYGS and LPSN: a database tandem for fast and reliable genome-based classification and nomenclature of prokaryotes. *Nucleic Acids Res* 2021;50:D801–D807:DOI: 10.1093/nar/gkab902.

3. **Meier-Kolthoff JP, Hahnke RL, Petersen J, Scheuner C, Michael V, *et al.*** Complete genome sequence of DSM 30083T, the type strain (U5/41T) of *Escherichia coli*, and a proposal for delineating subspecies in microbial taxonomy. *Stand Genomic Sci* 2014;9:2:DOI: 10.1186/1944-3277-9-2.

4. **Edgar RC**. MUSCLE: multiple sequence alignment with high accuracy and high throughput. *Nucleic Acids Res* 2004;32:1792–1797:DOI: 10.1093/nar/gkh340.

5. **Stamatakis A**. RAxML version 8: a tool for phylogenetic analysis and post-analysis of large phylogenies. *Bioinformatics* 2014;30:1312–1313:DOI: 10.1093/bioinformatics/btu033.

6. **Goloboff PA, Farris JS, Nixon KC**. TNT, a free program for phylogenetic analysis. *Cladistics* 2008;24:774–786:DOI: 10.1111/j.1096-0031.2008.00217.x.

7. **Pattengale ND, Alipour M, Bininda-Emonds ORP, Moret BME, Stamatakis A**. How Many Bootstrap Replicates Are Necessary? *J Comput Biol* 2010;17:337–354:DOI: 10.1089/cmb.2009.0179.

8. **Swafford DL**. PAUP*: Phylogenetic Analysis Using Parsimony (*and Other Methods). Version 4.0 b10. https://paup.phylosolutions.com/ (2002, accessed 3 June 2024).
